# *Mycobacterium tuberculosis* Uses Host Triacylglycerol to Accumulate Lipid Droplets and Acquires a Dormancy-Like Phenotype in Lipid-Loaded Macrophages

**DOI:** 10.1371/journal.ppat.1002093

**Published:** 2011-06-23

**Authors:** Jaiyanth Daniel, Hédia Maamar, Chirajyoti Deb, Tatiana D. Sirakova, Pappachan E. Kolattukudy

**Affiliations:** Burnett School of Biomedical Sciences, College of Medicine, University of Central Florida, Orlando, Florida, United States of America; University of New Mexico, United States of America

## Abstract

Two billion people are latently infected with *Mycobacterium tuberculosis* (*Mtb*). *Mtb*-infected macrophages are likely to be sequestered inside the hypoxic environments of the granuloma and differentiate into lipid-loaded macrophages that contain triacylglycerol (TAG)-filled lipid droplets which may provide a fatty acid-rich host environment for *Mtb*. We report here that human peripheral blood monocyte-derived macrophages and THP-1 derived macrophages incubated under hypoxia accumulate Oil Red O-staining lipid droplets containing TAG. Inside such hypoxic, lipid-loaded macrophages, nearly half the *Mtb* population developed phenotypic tolerance to isoniazid, lost acid-fast staining and accumulated intracellular lipid droplets. Dual-isotope labeling of macrophage TAG revealed that *Mtb* inside the lipid-loaded macrophages imports fatty acids derived from host TAG and incorporates them intact into *Mtb* TAG. The fatty acid composition of host and *Mtb* TAG were nearly identical suggesting that *Mtb* utilizes host TAG to accumulate intracellular TAG. Utilization of host TAG by *Mtb* for lipid droplet synthesis was confirmed when fluorescent fatty acid-labeled host TAG was utilized to accumulate fluorescent lipid droplets inside the pathogen. Deletion of the *Mtb triacylglycerol synthase 1* (*tgs1*) gene resulted in a drastic decrease but not a complete loss in both radiolabeled and fluorescent TAG accumulation by *Mtb* suggesting that the TAG that accumulates within *Mtb* is generated mainly by the incorporation of fatty acids released from host TAG. We show direct evidence for the utilization of the fatty acids from host TAG for lipid metabolism inside *Mtb*. Taqman real-time PCR measurements revealed that the mycobacterial genes *dosR*, *hspX, icl1, tgs1* and *lipY* were up-regulated in *Mtb* within hypoxic lipid loaded macrophages along with other *Mtb* genes known to be associated with dormancy and lipid metabolism.

## Author Summary

Two billion people are latently infected with *Mycobacterium tuberculosis* (*Mtb*). Cure and possible eradication of tuberculosis are limited by the lack of availability of any drug that can kill dormant *Mtb*. Understanding of the processes critical for dormancy and a reliable dormancy model suitable for high throughput screening of chemicals will help to discover drugs that can kill dormant *Mtb.* Storage of lipids for utilization as energy source is critically needed for dormancy. In the human lung, *Mtb*-infected macrophages are sequestered inside the hypoxic environments of the physical enclosure called granuloma in which *Mtb* becomes dormant. None of the currently used cell culture models of *Mtb* infection mimic this situation. We developed a model that mimics the environment inside the human granuloma by incubating *Mtb*-infected macrophages under hypoxia. We found that, under these conditions, macrophages accumulate lipid droplets and *Mtb* within these macrophages acquire a dormancy phenotype. We report how the pathogen inside the macrophages utilizes the host lipids to store lipids within the pathogen and acquire the hallmark traits of dormant *Mtb*. Thus, our novel model of *Mtb* dormancy may enable better understanding of the metabolic processes vital for the dormant pathogen and help to discover drugs that can kill latent pathogens.

## Introduction

One-third of the world population is latently infected with *Mycobacterium tuberculosis* (*Mtb*) and this vast reservoir is expected to contribute towards an increasing incidence of tuberculosis (TB) disease. The World Health Organization estimated recently that there were 11 million prevalent cases of the disease and 1.8 million deaths annually due to TB, including 0.5 million deaths in HIV-positive patients [1]. *Mtb*, the causative agent, is inhaled as an aerosol and enters the lung where it infects the alveolar macrophages and eludes host defenses. The primary immune response of the host controls bacillary multiplication and causes the pathogen to enter a state of dormancy and become phenotypically antibiotic tolerant leading to latent TB [2], [3], [4]. As a result of the host immune response, the pathogen is contained within the granuloma which is made up of infected macrophages surrounded by foamy lipid-loaded macrophages, mononuclear phagocytes and lymphocytes enclosed within a fibrous layer of endothelial cells [5], [6], [7]. *Mtb* can persist inside the host for decades until the host immune system is weakened and then reactivates to cause active disease [3].

It was established several decades ago that *Mtb* inside the host uses fatty acids as the major source of energy [8]. Isocitrate lyase (icl), which has been known to be a key enzyme of the glyoxylate cycle used by organisms that live on fatty acids [9], was shown to be vital for the pathogen's persistence inside the host demonstrating the critical role of fatty acids as an energy source for *Mtb* [10]. Based on the observation that fatty acids are normally stored as triacylglycerol (TAG) in the adipose tissues of mammals, seed oils of plants and as lipid inclusion bodies in prokaryotes for use as energy source during and after dormancy/ hibernation, TAG was postulated to be the storage form of energy for latent *Mtb* [11]. Intracellular lipid inclusion bodies were initially observed in *Mtb* more than six decades ago and were more recently detected in mycobacteria isolated from the sputum of TB patients [12], [13]. We showed that TAG accumulation is a critical event of *Mtb* dormancy and reported the discovery of *triacylglycerol synthase 1 (tgs1)* as the primary contributor to TAG synthesis within the pathogen and that the deletion of *tgs1* led to a nearly complete loss in TAG accumulation by *Mtb* under *in vitro* dormancy-inducing conditions [11], [14], [15]. Recent observations from other groups have shown that the *tgs1* gene is upregulated and TAG accumulates in dormant *Mtb* found in the sputum of TB patients and in the widespread, multi-drug resistant W/Beijing strain of *Mtb* [16], [17].

The source of fatty acids for synthesis of the TAG that accumulates as lipid droplets in the pathogen remains unknown. In humans with untreated pulmonary TB, caseous granulomas in the lungs were shown to contain lipid-loaded foamy macrophages which harbored acid-fast bacilli [7]. Such lipid-loaded macrophages which are found inside the hypoxic environment of the tuberculous granuloma contain abundant stores of TAG and are thought to provide a lipid-rich microenvironment for *Mtb* [5], [6]. Human macrophages cultured under hypoxia (1% O_2_) accumulate TAG in lipid droplets [18]. *Mtb*-infected human alveolar macrophages are most likely enclosed in a hypoxic environment within the granuloma where the pathogen becomes dormant. It was shown recently that tuberculous granulomas in guinea pigs, rabbits and non-human primates were hypoxic [19]. It is well recognized that nonpulmonary tissue oxygen concentrations within the human body are far below the oxygen concentration in ambient room air and the typical oxygen level in standard *in vitro* cell cultures is much higher than that encountered by macrophages inside the human body [20], [21]. Furthermore, the oxygen concentration in the phagosome of activated macrophages was shown to be lower than the extracellular oxygen concentration [22]. Dissemination of *Mtb* to distal sites such as the adipose tissue may also provide a TAG-enriched host environment for *Mtb* to go into dormancy [23]. We postulate that *Mtb* inside lipid-loaded macrophages might import fatty acids derived from host TAG to accumulate TAG inside the bacterial cell and provide evidence to support this hypothesis.

We infected human peripheral blood mononuclear cell (PBMC)-derived macrophages and THP-1 derived macrophages (THPM) with *Mtb* and incubated them under hypoxia (1% O_2_) in order to mimic the microenvironment within the human lung granuloma. We demonstrate that the macrophages accumulate lipid droplets under hypoxia. Using single and double isotope labeling methods to metabolically label the host TAG, we determined that *Mtb* imports fatty acids released from host TAG to accumulate TAG within the bacterial cell. Host fatty acids were incorporated intact into *Mtb* TAG. We also show that host TAG that was metabolically labeled with a fluorescent fatty acid was imported by *Mtb* and accumulated as fluorescent lipid droplets within the bacterial cell. Deletion of *tgs1* resulted in a drastic decrease in radiolabeled and fluorescent TAG accumulation within *Mtb* inside THPM thereby revealing that synthesis of TAG within the pathogen from fatty acids released from host TAG constitutes the major pathway of TAG accumulation by *Mtb* inside the host. We demonstrate that *Mtb* cells within lipid-loaded macrophages accumulate lipid droplets containing TAG, lose acid-fast staining and become phenotypically resistant to the two frontline antimycobacterial drugs, rifampicin (Rif) and isoniazid (INH), all of which are thought to be indicative of the dormant state of the pathogen [2], [11], [15], [24], [25]. Taqman real-time PCR analysis of gene transcripts of *Mtb* recovered from lipid-loaded macrophages revealed that genes thought to be involved in dormancy and lipid metabolism were upregulated within the pathogen.

## Results

### Macrophages accumulate triacylglycerol in lipid droplets under hypoxia

**Figure 1:**
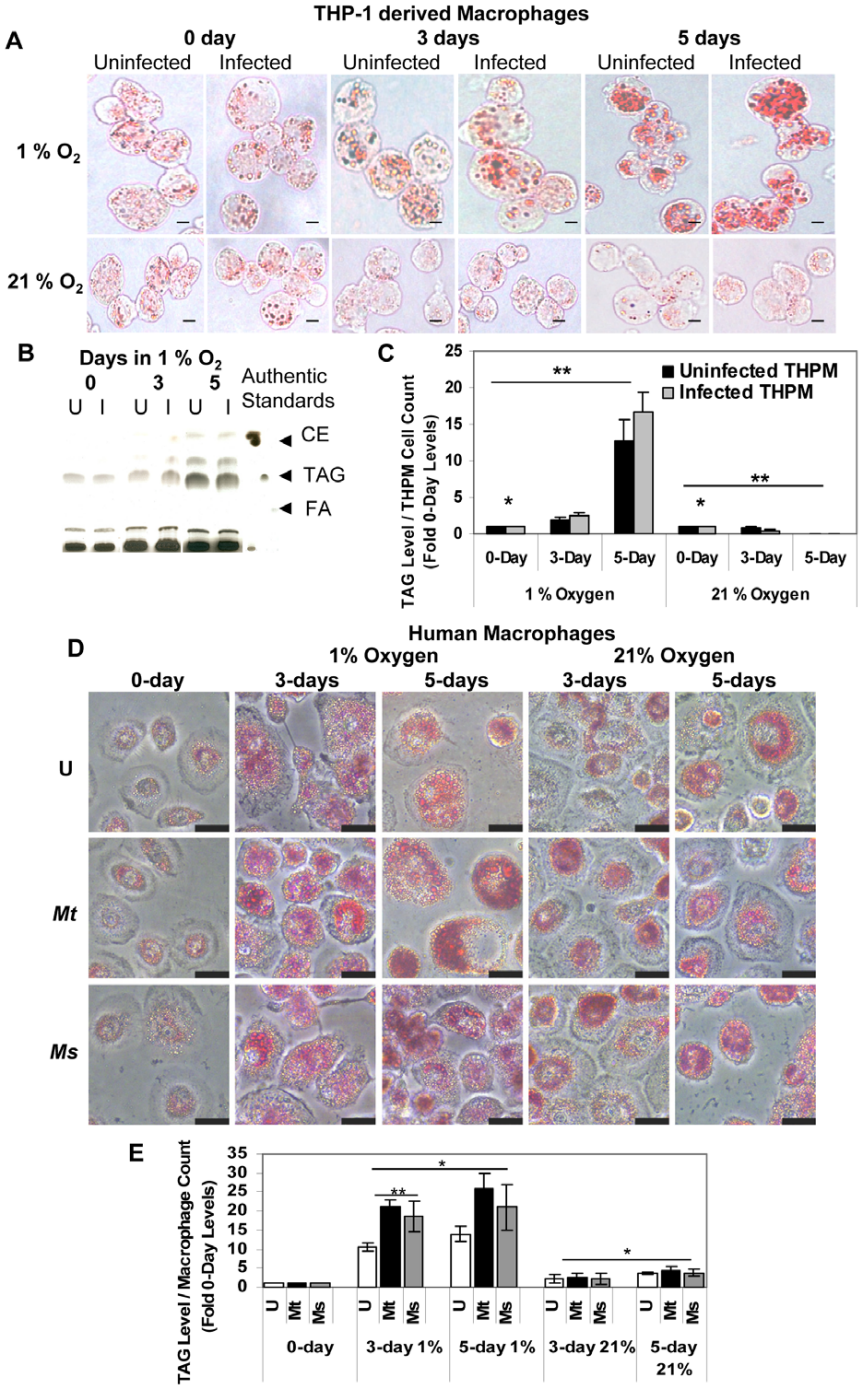
Macrophages under hypoxia accumulate Oil Red O staining lipid droplets containing triacylglycerol. **A,** Oil Red-O stained lipid droplets increase in THPM under 1 % O_2_ independent of *Mtb* infection but do not increase in THPM incubated under 21% O_2_. THPM were infected with *Mtb* at an MOI of 0.1 and incubated under hypoxia or normoxia as described in Methods. At each time-point, trypsinized THPM were fixed with 4% paraformaldehyde and stained with Oil Red-O. Uninfected THPM were incubated under identical conditions. Scale bar, 10 µm. **B**, Silica-TLC of lipid extracts from uninfected (U) and infected (I) hypoxic THPM. The lipid extracts were resolved on silica-TLC and visualized by dichromate-sulfuric acid charring as described in Methods. Relative migrations of authentic standard cholesteryl ester (CE), TAG and fatty acid (FA) are shown. **C**, TAG levels in THPM increase under hypoxia but not under normoxia. TAG band intensities on TLC plates were normalized to the respective viable THPM cell counts and represented as fold of 0-day levels. 0-day levels were assigned an arbitrary value of 1. Data is represented as average ± SD from a representative experiment (n = 3). *, Statistically insignificant difference (p>0.05) between uninfected and infected THPM; **, statistically significant difference (p<0.05) between 1% O_2_ and 21% O_2_ samples. **D**, Oil Red-O stained lipid droplets increase in number and size in human PBMC-derived macrophages incubated under 1% O_2_ than in those incubated under 21% O_2_. U, *Mt*, *Ms* represent uninfected, *Mtb*-infected and *M. smegmatis*-infected macrophages respectively. Macrophages were infected at an MOI of 0.1 and incubated under hypoxia or normoxia as described in Methods. Scale bar, 20 µm. **E**, Hypoxia strongly induces TAG accumulation in human macrophages. *M. smegmatis*-infected hypoxic macrophages accumulated lower levels of TAG than *Mtb*–infected cells. TAG band intensities were determined from TLC analysis of macrophage total lipid extracts and normalized to viable macrophage cell counts. Data is represented as average ± SD from a representative experiment (n = 3). *, Statistically significant difference (p<0.05) between 1% O_2_ and 21% O_2_ samples **, statistically significant difference (p<0.05) between uninfected and infected macrophages under hypoxia.

Human alveolar macrophages, in which *Mtb* multiplies, probably reach a hypoxic environment within the granuloma, in which the pathogen goes into a latent state. Such macrophages are likely to be lipid-loaded as a consequence of hypoxia and *Mtb* infection, both of which have been reported to induce lipid accumulation in macrophages *in vitro* [6], [18], [26]. It is well known that nonpulmonary tissue oxygen concentrations within the human body are much lower than the oxygen level in ambient air and that caseous granulomas in rabbits are hypoxic [19], [21]. In order to mimic the hypoxic microenvironment within the granuloma, we infected human PBMC-derived macrophages and THPM with low numbers of *Mtb* (MOI 0.1 to 5) and incubated them under 1% O_2_, 5% CO_2_. About 3% of the host cells were infected at MOI 0.1 as determined by the CFUs recovered from the infected host cells after 4 h infection. Oil Red O-staining lipid bodies increased upto 5 days in *Mtb*-infected macrophages as well as uninfected macrophages incubated under 1% O_2_ (Figure 1A, D). In contrast, lipid bodies increased moderately in macrophages incubated under 21% O_2_ (Figure 1A, D). TAG was the major lipid that accumulated in THPM lipid droplets under hypoxia and maximal levels were reached by day 5 (Figure 1B and C). Longer incubations resulted in greater loss of THPM from the adhered monolayer (data not shown). TAG accumulation in lipid bodies was also strongly induced under hypoxia in human PBMC-derived macrophages (Figure 1 D, E). Lipid droplets containing TAG increased greatly in size and number with time of culturing under hypoxia but only moderately under normoxia, and when normalized to viable macrophage cell counts, it was observed that TAG levels in hypoxic macrophages were much higher than that in normoxic macrophages. There were considerable differences in lipid body formation between macrophages in the same population. Since the photomicrographs showing selected fields of Oil Red O-stained macrophages do not adequately represent the TAG levels in the whole macrophage population, we relied on the analysis by thin-layer chromatography (TLC) of the TAG levels in the lipid extracts from the total population.

Since it was reported earlier that oxygenated mycolic acids, which are found only in virulent mycobacteria but absent in the non-virulent *Mycobacterium smegmatis*, were necessary for lipid body formation in macrophages under normoxic conditions [6], we tested whether such a mechanism may be involved in lipid body formation in human macrophages under hypoxia. Our observations indicate that macrophages accumulate TAG upon hypoxic stress alone since uninfected macrophages accumulated lipid droplets containing TAG to significantly higher levels under hypoxia than under normoxia (Figure 1D,E). We observed that the levels of TAG were slightly lower in *M. smegmatis*-infected macrophages than in *Mtb*-infected macrophages under hypoxia. However, this difference was not significant under normoxic conditions. Macrophages obtained from human PBMCs after differentiation for 7 days contained varying levels of small lipid droplets between different individual donors suggesting donor-to-donor variations in macrophage characteristics. Moreover, the increase in lipid body size and number under hypoxia varied by different degrees between donors.

### *Mtb* within hypoxic lipid-loaded macrophages accumulates intracellular TAG mainly by the action of *tgs1* gene product

**Figure 2:**
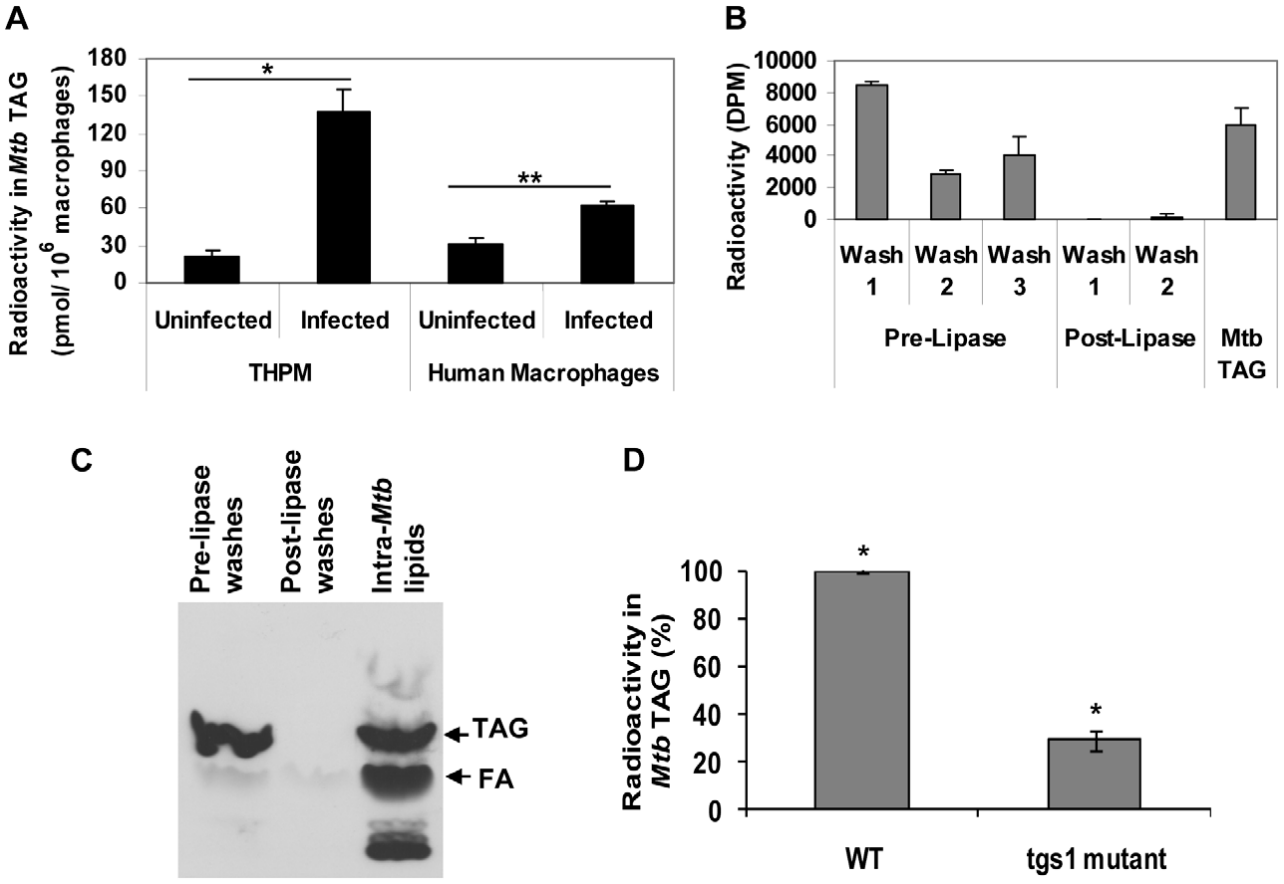
*Mtb* within hypoxic lipid-loaded macrophages accumulates intracellular triacylglycerol predominantly by the action of *tgs1*. **A**, *Mtb* recovered from radiolabeled macrophages accumulates intracellular TAG. THPM or human PBMC-derived macrophages labeled with^14^C-oleate were infected with *Mtb* at an MOI of 5 bacilli per host cell and incubated under 1% O_2_ for 3 days after which *Mtb* cells (3500 x g pellet) were recovered and *Mtb* TAG levels measured as described in Methods. Data is represented as average ± SD from a representative experiment (n = 3). *, Statistically significant difference (p<0.005) between uninfected and infected THPM samples. **, Statistically significant difference (p<0.05) between uninfected and infected human macrophage samples. **B**, *Mtb* accumulates intracellular radiolabeled TAG synthesized from host-derived fatty acids. Radioactivity measurements validate the efficacy of the detergent washes and lipase treatments in removing contaminating radiolabel from the exterior surface of *Mtb* prior to lipid extraction. TAG extracted from within *Mtb* contains significant radioactivity. Average ± SD from a typical experiment is shown (n = 3). **C**, *Mtb* accumulates intracellular TAG derived from extracellular TAG. *Mtb* grown *in vitro* to mid log-phase in Middlebrook 7H9 medium was metabolically labeled with 10 µCi ^14^C-triolein for 2 h under aerobic conditions. *Mtb* cells were washed with detergent and treated with lipase as described in Methods. *Mtb* lipids and lipids in washes were resolved on silica-TLC and autoradiogram of TLC plate is shown. Relative migration of authentic standard TAG and fatty acid (FA) are indicated by arrows. **D**, *tgs1* gene product is the main contributor to TAG synthesis within *Mtb* inside hypoxic THPM. *Mtb* wild-type (WT) or Δ*tgs1* mutant were used to infect THPM labeled with ^14^C-oleate at an MOI of 5.0 and were recovered after 3 days in 1% O_2_. Radioactivity in *Mtb* TAG was determined as described in Methods. Triplicate measurements were used to calculate average ± SD values (n = 4); *, Statistically significant difference (p<0.005) between TAG levels in WT and Δ*tgs1*.

It has been well established by our group and others that intracellular TAG is accumulated inside *Mtb* under *in vitro* dormancy-inducing conditions and in *Mtb* from sputum of human TB patients and that the *tgs1* gene product of *Mtb* is a major contributor to this process [11], [13], [14], [15], [16], [17]. In order to determine whether *Mtb* cells inside lipid-loaded macrophages utilized host lipids for accumulating TAG inside the bacterial cell, we radiolabeled macrophages with [1–^14^C]oleate (10 µCi/ 7×10^6^ THPM or 10 µCi/ 4×10^6^ human PBMC-derived macrophages) under 1% O_2_, 5% CO_2_ for 24 h prior to infection with *Mtb* (5 bacilli per macrophage). The infected macrophages were then incubated under 1% O_2_, 5% CO_2_ for 3 days. *Mtb* were recovered by lysing the host cells and centrifuging the lysate at 3500 x g. As described in Methods, the 3500 x g pellets containing *Mtb* were washed thoroughly with mild detergent to remove host TAG adhering to the outside of the *Mtb* cells and any remaining host TAG was removed by enzymatic hydrolysis by TAG lipase which was followed by further detergent washes. The 3500 x g pellet of uninfected host cell lysate was used as a control for background TAG levels. We observed that radioactivity in TAG in the 3500 x g pellets of the infected human PBMC-derived macrophages and THPM was significantly higher than background controls suggesting that *Mtb* inside the host cells was utilizing the radiolabeled host lipids to accumulate TAG within the bacterial cell (Figure 2A). Moreover, TAG levels increased with time in live *Mtb* cells during infection of THPM under hypoxia but not in heat-killed *Mtb* cells indicating that intracellular TAG accumulation required active processes in live *Mtb* (data not shown).

In order to determine whether the TAG that accumulates in *Mtb* cells inside radiolabeled macrophages is indeed intra-bacterial TAG, we labeled 7×10^6^ THPM with about 18×10^6^ dpm [9,10-^3^H]oleic acid (per data point) under 1% O_2_ for 24 h prior to infection with *Mtb*. About 17×10^6^ dpm (98%) of the radiolabeled oleic acid was taken up by the host cells under these conditions and incorporated into TAG. The radioactivity in THPM TAG (1.5×10^6^ dpm) accounted for nearly 32% of total macrophage lipids (4.7×10^6^ dpm). The radiolabeled THPM were infected with *Mtb* at an MOI of 5.0 and incubated 3 days under 1% O_2_. As shown in Figure 2B, the detergent washes and lipase treatment were effective in removing radioactive material from the exterior of the *Mtb* cells. Extraction and TLC analysis of lipids from the washed *Mtb* cells revealed that radiolabeled macrophage-derived fatty acids were indeed imported and stored as TAG inside *Mtb* (Figure 2B, *Mtb* TAG).

In order to determine whether *Mtb* is capable of importing fatty acids derived from TAG outside the bacterial cell and confirm the above findings which suggested that *Mtb* utilized radiolabeled fatty acids from host TAG to accumulate radiolabeled TAG inside the bacterial cell, we incubated *Mtb* with radiolabeled TAG in culture medium. A mid-log phase culture of *Mtb* was labeled with ^14^C-triolein for 2 h under aerobic conditions. The *Mtb* cells were then washed with detergent and treated with lipase to remove any radiolabeled TAG that may be adhered to the extracellular surface of the *Mtb* cells. Intracellular *Mtb* lipids and lipids in the washes prior to and after lipase treatment were resolved on silica-TLC. The autoradiogram shown in Figure 2C reveals that the washes combined with lipase treatment were effective in removing TAG adhered to the exterior of the *Mtb* cell. Post-lipase washes had almost no TAG. Bacterial lipids were not removed by the lipase treatment and washes. Most importantly, the lipid extract from the washed *Mtb* cells showed that TAG is stored inside the bacterial cell (Figure 2C, Intra-*Mtb* lipids). It is also evident that the radiolabeled fatty acids imported from extracellular TAG into *Mtb* are utilized by the bacteria for synthesis of other *Mtb* lipids.

In order to determine whether the major triacylglycerol synthase gene of *Mtb* (*tgs1*) is involved in TAG accumulation inside the bacilli within hypoxic lipid-loaded THPM, we infected THPM radiolabeled with oleate with *Mtb* wild-type or *Mtb* Δ*tgs*1 mutant and incubated the infected host cells under hypoxia for 3 days. Deletion of *tgs1* resulted in a severe reduction, but not a complete loss, of radiolabeled TAG accumulation by *Mtb* inside THPM (Figure 2D). This finding suggested that the TAG accumulated inside *Mtb* within lipid-loaded macrophages was synthesized mainly by re-esterifying host lipid-derived fatty acids into TAG by *Mtb tgs1* gene product. The TAG that accumulates in the *tgs1* mutant is probably generated by the other *Mtb tgs* gene products.

### *Mtb* accumulates intracellular lipid droplets containing TAG derived from fluorescent fatty acid-labeled host TAG

**Figure 3:**
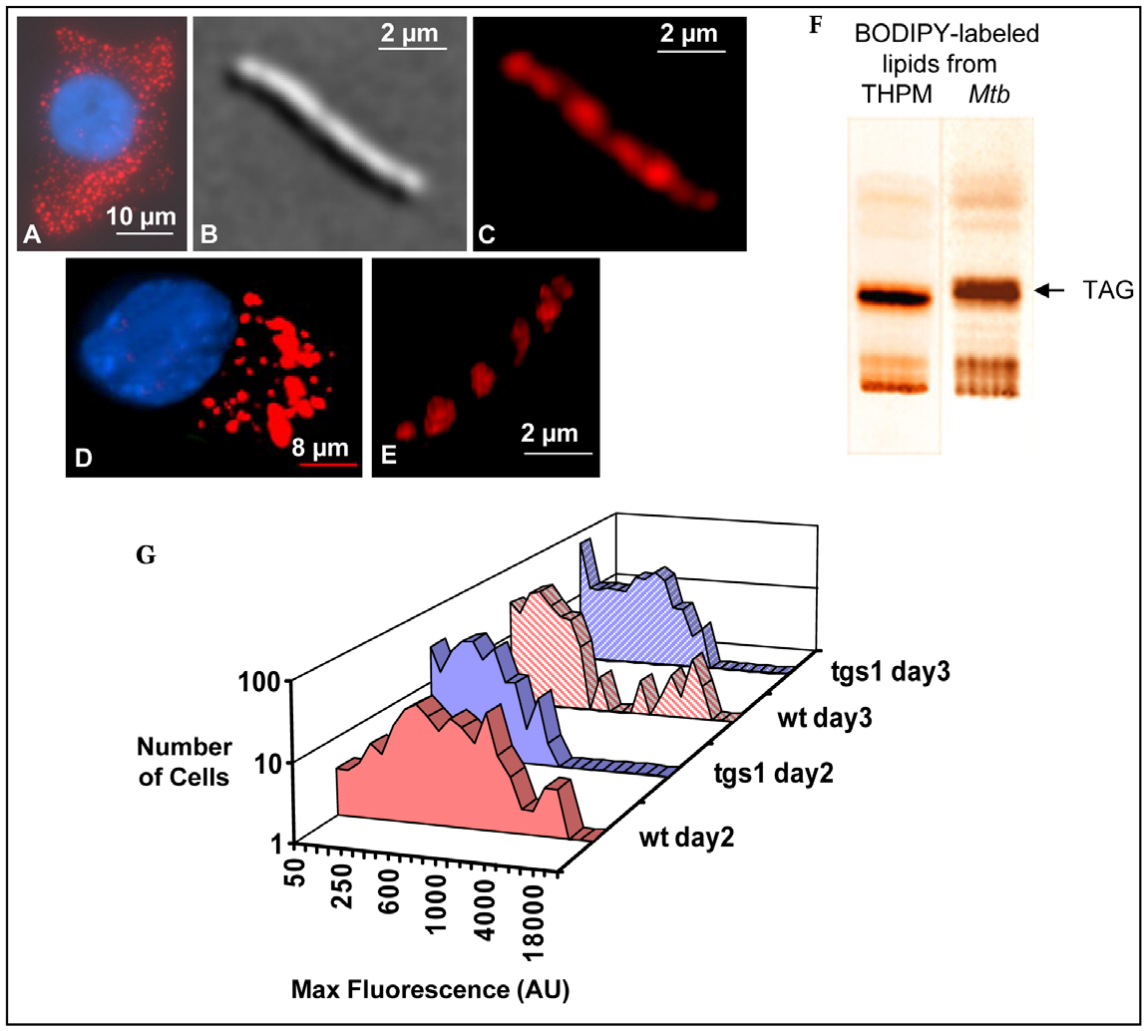
*Mtb* mobilizes macrophage triacylglycerol labeled with fluorescent fatty acid and accumulates fluorescent intracellular lipid droplets. THPM were allowed to metabolically incorporate the fluorescent fatty acid BODIPY 558/568 C_12_ for 24 h and unincorporated label was removed by washing prior to infection with *Mtb*. **A**, Intact lipid-loaded macrophage viewed under TRITC filter showing lipid droplets (red) metabolically labeled with fluorescent fatty acid. Nucleus stained with DAPI and overlay shown. **B**, Differential interference contrast image of *Mtb* recovered from THPM and **C**, Image of the same *Mtb* cell viewed with TRITC filter showing fluorescent BODIPY fatty acid-labeled lipid droplets within the pathogen. **D**, Snapshot of the 3D image of an intact lipid-loaded macrophage. The 3D image is constructed from image stacks taken with the appropriate filter sets for each stain and overlayed. **E**, Snapshot of the 3D image (obtained as described for the THPM, with only the TRITC filter set) of an *Mtb* cell recovered from THPM 6 days after infection showing lipid droplets within the pathogen. **F**, TLC showing that fluorescent TAG is the predominant lipid in both THPM and in *Mtb* within THPM. THPM were metabolically labeled with BODIPY fatty acid for 24 h under 1% O_2_ and then infected with *Mtb* and incubated for a further 36 h. A small aliquot of THPM lipids and most of the lipids from *Mtb* were applied to the TLC plate. After silica-TLC with hexane: diethyl ether: formic acid (40∶10∶1) as the solvent, the plate was imaged under UV illumination with Texas Red filter. **G**, Fluorescence maximum intensities in individual *Mtb* cells of the WT (red) and the *tgs1* (blue) strains showing that lipid-droplet accumulation inside *Mtb* is impaired in the absence of *tgs1*. Cells of both strains were recovered from THPM pre-labeled with the BODIPY 558/568 C_12_ and infected at an MOI of 0.25. Measurements of fluorescence intensities were performed as described in Methods. Values from a representative experiment shown (n = 3). wt, wild type *Mtb*; *tgs1*, Δ*tgs1* mutant; AU, arbitrary units.

In order to obtain an independent confirmation of our findings above that indicated the import of host TAG-derived fatty acids by *Mtb* and their subsequent accumulation as TAG within the bacterial cell, we metabolically labeled host TAG with the fluorescently-tagged fatty acid, BODIPY 558/568 C_12_. The fluorescent fatty acid was incorporated into the lipid bodies accumulating in THPM under 1% O_2_ (Figure 3A,D). When these THPM containing fluorescent lipid bodies were infected with *Mtb* and incubated under 1% O_2_, the pathogen became loaded with well defined, highly fluorescent lipid bodies inside the cells (Figure 3B,C,E). Infection of THPM at MOIs of 0.1 and 0.25 yielded similar results and we selected an MOI of 0.25 in order to have higher numbers of *Mtb* cells for statistical purposes. Optical cross-sectioning of the 3D image of *Mtb* containing fluorescent fatty acid-labeled lipid droplets revealed that the lipid droplets were intracellular suggesting that the pathogen generates intracellular TAG using fatty acids derived from host TAG (Figure 3E). TLC analysis of lipids extracted from fluorescent fatty acid-labeled THPM and *Mtb* recovered from such THPM revealed that TAG was the predominant lipid in both (Figure 3F). Thus, *Mtb* imports the fluorescently labeled fatty acids derived from the host TAG and accumulates fluorescent, intracellular lipid droplets comprised mostly of TAG.

Microscopic measurement of fluorescence in a population of 250 individual *Mtb* cells recovered from THPM showed that after 2 days of infection, most of the *Mtb* cells contained lipid droplets with intermediate levels of fluorescence and a smaller number contained intensely fluorescent lipid droplets (Figure 3G). At day 3, the cellular distribution of fluorescence intensities was clearly bimodal, with about 8% of the cells in a high-fluorescence-level subpopulation. Most of these cells in this subpopulation contained well defined lipid bodies. This fraction of the population was reduced to about 1% in mutant *Mtb* cells lacking *tgs1*. Overall, the fluorescent TAG accumulation was severely reduced in the Δ*tgs1* mutant at all time points compared to the wild type. These results suggest that *tgs1* plays a significant role in the synthesis of TAG within *Mtb* from host TAG-derived fatty acids.

### *Mtb* TAG is synthesized using fatty acids released from host TAG

**Figure 4:**
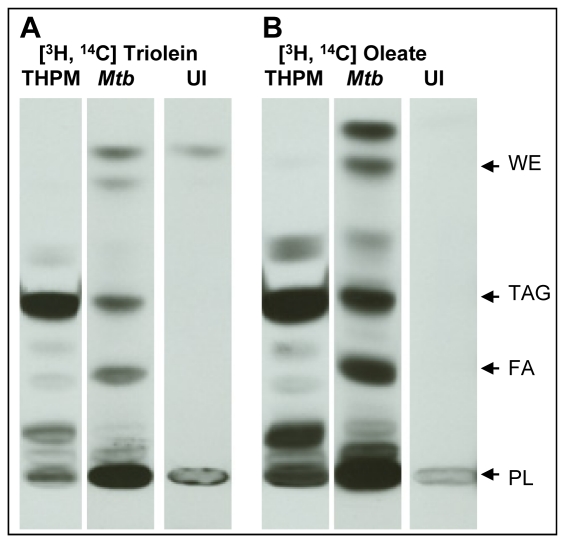
*Mtb* inside lipid-loaded macrophages imports host fatty acids for storage as TAG. THPM were double isotope labeled with triolein [glycerol-1,2,3-^3^H, carboxyl-1-^14^C] (**A**) or oleic acid [9,10-^3^H, 1-^14^C] (**B**) for 24 h in 1% O_2_, 5% CO_2_ prior to *Mtb* infection at an MOI of 5. All 3500 x g pellets were detergent-washed and lipase-treated prior to lipid extraction as described in Methods. Total lipid extracts of dual isotope-labeled THPM and *Mtb* recovered from THPM were analyzed at 72 h post-infection. Lipids were resolved on silica TLC using hexane: diethyl ether: formic acid, 40∶10∶1 by volume, as solvent system and autoradiograms are shown. 3500 x g pellets of uninfected host cell lysates show no cross-contamination with host TAG (Lanes “UI” in **A, B**). Arrows indicate the relative positions of authentic internal lipid standards. UI, Uninfected background control, WE, wax esters; TAG, triacylglycerol; FA, fatty acids; PL, polar lipids.

To examine whether *Mtb* inside THPM imported intact host TAG or fatty acids from host TAG, we metabolically labeled THPM with dual isotope labeled triolein [glycerol-1,2,3–^3^H, carboxyl-1-^14^C] under 1% O_2_ for 24 h prior to infection with *Mtb* at an MOI of 5.0. If *Mtb* hydrolyzed host TAG and the fatty acids were used for TAG synthesis within *Mtb,* the ^3^H:^14^C ratio of the TAG from *Mtb* recovered from THPM should be different and probably less than that of the host TAG. If host TAG was taken up intact by the pathogen, the isotopic ratio of TAG in the pathogen should be the same as that of host TAG. *Mtb* were recovered from THPM after 3 days in 1% O_2_, washed with mild detergent and treated with TAG lipase to remove contaminating extracellular host TAG prior to lipid extraction. Uninfected background controls did not contain TAG thereby demonstrating the efficacy of detergent washes and lipase treatment in removing host TAG contamination in the 3500 x g pellets containing *Mtb* (Figure 4, lanes UI). TLC analysis showed that the radioactivity in the total lipid extracts of THPM was primarily in TAG and that *Mtb* accumulated radiolabeled TAG inside THPM (Figure 4A and Table 1). Dual isotope-labeled TAG was purified from total lipid extracts of host (3500 x g supernatant) and *Mtb* (3500 x g pellet) by silica-TLC and the ^3^H:^14^C ratios were determined. As indicated in Table 1, the ratio of *Mtb* TAG was significantly lower than THPM TAG suggesting that TAG found in *Mtb* inside THPM was synthesized mainly from fatty acids released from host TAG. The *Mtb* cells inside THPM also accumulated wax esters, albeit to a lower extent (Figure 4A and B).

**Table 1:** Fatty acids derived from host triacylglycerol are imported by *Mtb* inside lipid-loaded macrophages.

Triolein [glycerol-1,2,3-^3^H, carboxyl-1-^14^C] Dual Isotope Labeling						
	^3^H: ^14^C DPM Ratios		Radioactivity as Percent of Total Lipids			
	Experiment 1	Experiment 2	Experiment 1		Experiment 2	
			^3^H	^14^C	^3^H	^14^C
THPM TAG	0.23±0.025	0.33±0.032	56±1	74±6	63±10	54±1
*Mtb* TAG	0.11±0.006	0.18±0.012	5±1	11±2	3±1	6±1

THPM metabolically labeled with double isotope labeled triolein [glycerol-1,2,3-^3^H, carboxyl-1-^14^C] under 1% O_2_ for 24 h were infected with *Mtb* at an MOI of 5.0. *Mtb* were recovered from THPM after 3 days in 1% O_2_, and contaminating host TAG was removed prior to lipid extraction as described in Methods. TAG was purified from the respective total lipid extracts and the ratios of ^3^H and ^14^C radioactivities (dpm) were determined. The radioactivities in TAG are expressed as percentages of the radioactivities in the respective total lipid extracts. Average and standard deviation values were calculated from triplicate samples within each experiment.

As can be clearly seen in the TLC analyses of the lipids in Figure 4, it is evident that the lipid profile of *Mtb* recovered from radiolabeled macrophages (lanes “*Mtb*” in Figure 4A and B), is markedly different from that of the host cells (lanes “THPM” in Figure 4A and B) and uninfected background controls (lanes “UI” in Figure 4A and B). Within the *Mtb* cell, the radiolabel was found distributed among TAG, fatty acids (breakdown products of TAG), polar lipids (synthetic products incorporating fatty acids) and wax esters (storage lipids containing fatty acids). Thus, at the time of recovery of *Mtb* from the host cells, the *Mtb* cells were in the process of metabolizing the radiolabeled fatty acids derived from the host.

### Host TAG-derived fatty acids are incorporated directly into *Mtb* TAG

In order to determine whether fatty acids released from host TAG were incorporated intact into TAG, we metabolically labeled THPM with [9,10–^3^H, 1–^14^C]oleic acid and infected them with *Mtb* at an MOI of 5.0. TLC analysis revealed that the radioactivity in THPM lipids was primarily in TAG and that the total lipid profiles of host and *Mtb* were markedly different (Figure 4B, Table 2). If the host TAG-derived fatty acids were catabolized to acetate which was then used for fatty acid synthesis within *Mtb*, isotopic ratio of *Mtb* TAG should indicate a loss of ^3^H. We found that the ^3^H:^14^C ratio of *Mtb* TAG was nearly identical to THPM TAG indicating that host TAG-derived fatty acids were incorporated intact into *Mtb* TAG (Table 2).

**Table 2:** Host triacylglycerol-derived fatty acids imported by *Mtb* are incorporated intact into *Mtb* triacylglycerol.

Oleic acid [9,10-^3^H, 1-^14^C] Double Isotope Labeling Experiment			
	^3^H: ^14^C DPM Ratios	Radioactivity as Percent of Total Lipids	
		^3^H	^14^C
THPM TAG	0.47±0.01	32±6	51±8
*Mtb* TAG	0.56±0.01	5±1	6±0

	Percent Resistance of *Mtb* to	
	Rifampicin (5 µg/ml)	Isoniazid (0.8 µg/ml)
***Mtb*** ** inside THPM under 1 % oxygen**		
**Days in 1 % oxygen**		
(including 2 days with antibiotic)		
**2**	4±2	27±5
**5**	8±1	49±1
**7**	8±2	26±2
***Mtb*** ** inside human PBMC-derived macrophages under 1 % oxygen**		
**Days in 1 % oxygen**		
(including 2 days with antibiotic)		
**5**	16±5	55±12
**7**	18±2	43±15
***Mtb*** ** inside human PBMC-derived macrophages under 21 % oxygen**		
**Days in 21 % oxygen**		
(including 2 days with antibiotic)		
**2**	2±1	0.5±0.2
**5**	6±2	2±0.5
**7**	4±1	0.5±0.1

Double isotope labeled oleic acid [9,10-^3^H, 1-^14^C] was used for metabolically labeling THPM for 24 h under 1% O_2_. After infection of THPM at an MOI of 5.0 for 3 days, *Mtb* were recovered and contaminating host TAG removed prior to lipid extraction. TAG was purified from the respective total lipid extracts by silica-TLC and the ratios of ^3^H and ^14^C radioactivities (dpm) were determined as described in Methods. The radioactivities in TAG are expressed as percentages of the radioactivities in the respective total lipid extracts. Data from a typical experiment are shown as average and standard deviation values calculated from triplicate samples.

### Fatty acid composition of *Mtb* TAG is nearly identical to host TAG

**Figure 5:**
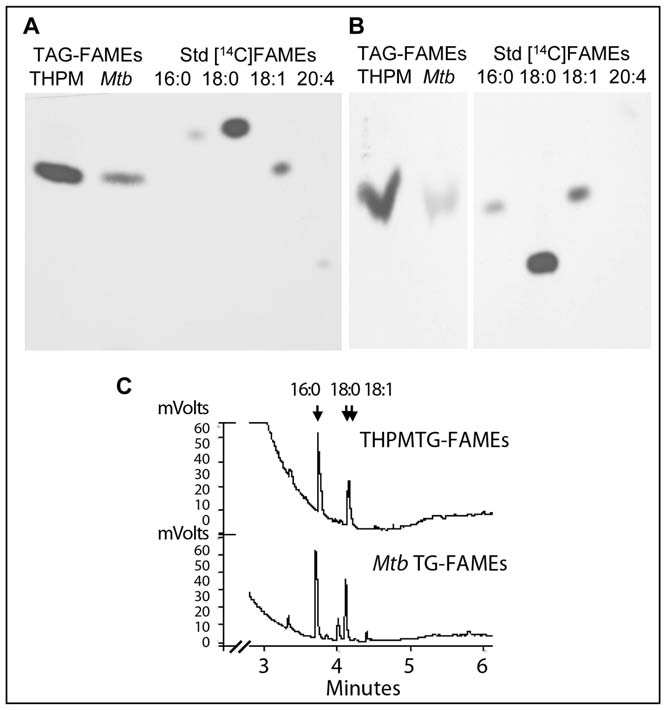
Fatty acid composition analysis confirms that *Mtb* incorporates host TAG-derived fatty acids directly into TAG. **A** and **B**, Macrophage TAG labeled with [^14^C]oleate is utilized by *Mtb* for TAG accumulation. **A**, AgNO_3_-TLC of methyl esters of fatty acids (FAMEs) prepared from TAG of *Mtb*-infected macrophages (lane 1, from left) and TAG from *Mtb* recovered from such macrophages (lane 2). **B**, Reversed-phase TLC analysis of FAMEs prepared from macrophage TAG (lane 1) and *Mtb* TAG (lane 2). Autoradiograms of the TLC plates with authentic ^14^C-labeled C16:0, C18:0, C18:1 and C20:4 FAMEs are shown. The AgNO_3_-TLC and reversed-phase TLC show that ^14^C-oleic acid is incorporated into THPM TAG which is utilized to accumulate [^14^C]oleate-labeled TAG inside *Mtb*. **C**, FAMEs prepared from THPM and *Mtb* TAG analyzed using a Varian CP-TAP CB capillary column attached to a Varian CP-3900 gas chromatograph under a temperature control program. *Mtb* TAG FAMEs are identical to THPM TAG FAMEs except for very long-chain derivatives seen only in the TAG from the pathogen.

We compared the fatty acid compositions of [1-^14^C]oleic acid-derived THPM TAG and TAG of *Mtb* recovered from THPM in order to obtain additional confirmation of the import of host fatty acids into *Mtb* TAG. By resolving the fatty acid methyl esters of the THPM and *Mtb* TAG on argentation-TLC (Figure 5A) and reversed-phase silica-TLC (Figure 5B), we found that all of the ^14^C in TAG from THPM and from *Mtb* isolated from THPM was found in oleic acid suggesting that host TAG-derived fatty acids were being incorporated into the TAG that accumulated within *Mtb* (Figure 5A,B).

The fatty acid composition of unlabeled host and *Mtb* TAG was determined by purifying and analyzing the fatty acid methyl esters derived from TLC-purified TAG by capillary gas chromatography. The fatty acid composition of the TAG from the pathogen was nearly identical to that of the host TAG. C16:0, C18:0 and C18:1 fatty acids were the dominant components in both the pathogen and the host (Figure 5C). Longer chain saturated fatty acids (C_24_, C_26_ and C_28_) that were present in very low amounts in the pathogen TAG were absent in the host TAG. We conclude that the TAG that accumulated in the pathogen consists predominantly of fatty acids derived from the host TAG.

### *Mtb* replication is severely inhibited inside hypoxic lipid-loaded macrophages

**Figure 6:**
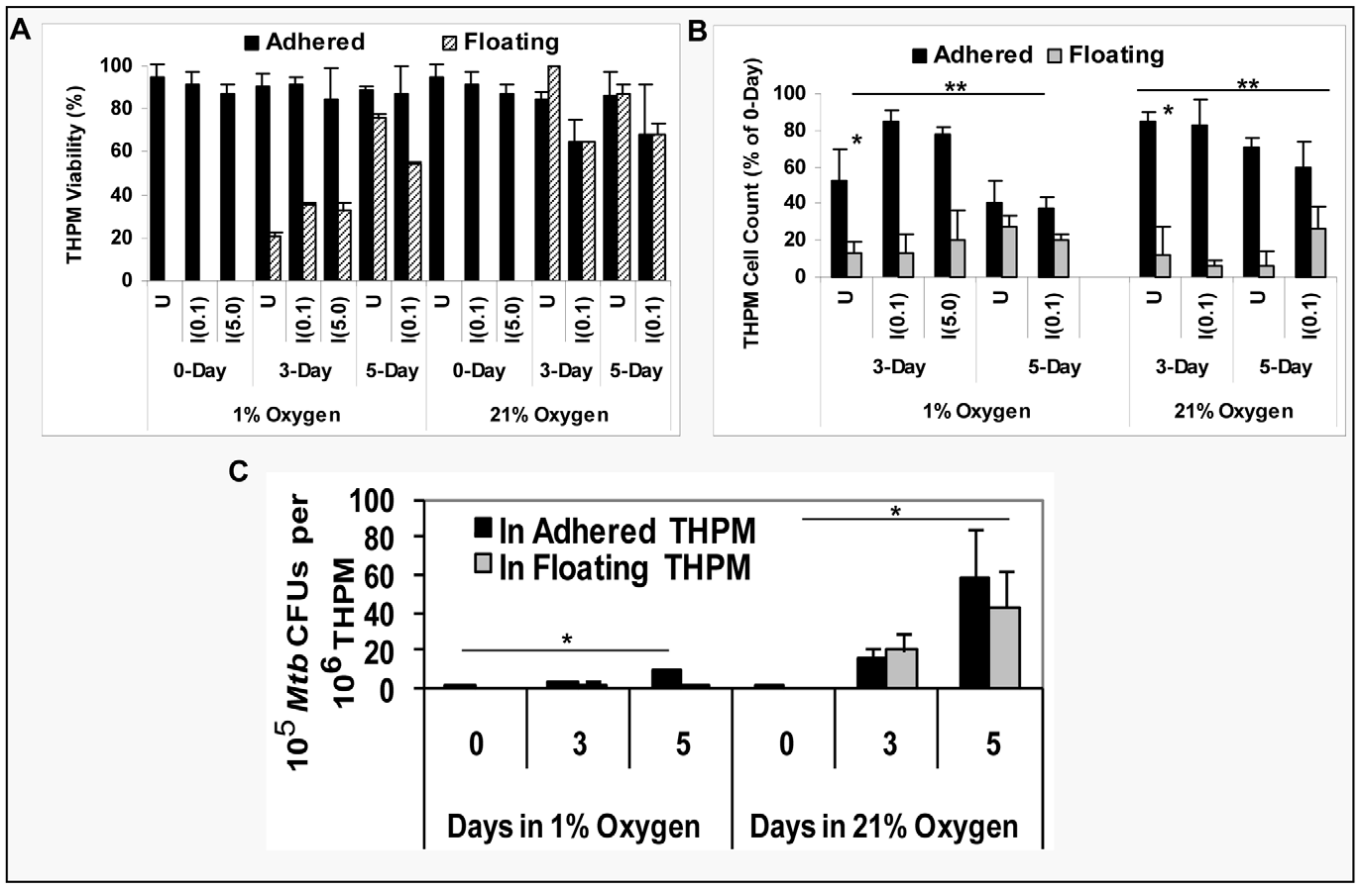
*Mtb* replicates slowly inside hypoxic lipid-loaded macrophages. A and **B**, THPM incubated under hypoxia are viable hosts for *Mtb*. Uninfected (U) and infected (I) cells (MOI 0.1 or MOI 5.0) were incubated in either 1% O_2_ or 21% O_2_. THPM cell viabilities (**A**) and cell counts (**B**) were determined for the floating and adhered THPM populations. Data from triplicate measurements presented as average ± SD (n = 3). In **B**; *, statistically significant differences (p<0.005) between adhered vs floating populations; **, statistically insignificant differences (p>0.05) between 1% vs 21% incubations;. **C**, *Mtb* replication inside hypoxic THPM is severely curtailed in contrast to normoxic THPM. THPM were infected at an MOI of 0.1 and incubated under 1% O_2_ or 21% O_2_. At 0, 3, 5-days, *Mtb* CFUs were determined by agar plating. *Mtb* CFUs were normalized to THPM numbers. Data from triplicate measurements presented as average ± SD (n = 3); *, statistically significant differences (p<0.05) between 1% O_2_ vs. 21% O_2_ incubations.

We assessed host cell numbers and viability in uninfected and infected THPM under hypoxia and normoxia and conclude that THPM cells incubated under 1% O_2_ are viable hosts for *Mtb* for upto 5 days at an MOI of 0.1 and upto 3 days at an MOI of 5. As determined by trypan blue dye exclusion method, THPM cell viability was about 90% in both cases (Figure 6A). At day 3 under 1% O_2_, about 85% of the original THPM population infected with *Mtb* at an MOI of 0.1 remained adhered as a monolayer and 80% of the THPM infected at an MOI of 5.0 remained adhered (Figure 6B). By day 5 under 1% O_2_, about 45% of the original *Mtb*-infected THPM population remained adhered as a monolayer loaded with lipid droplets after infection at an MOI of 0.1 (Figure 6B). Nearly half the host cells had perished under hypoxia and *Mtb* infection. Of the THPM incubated under 21% O_2_ after infection with *Mtb* at an MOI of 0.1, 80% remained adhered on day 3 but only 40% by day 5 (Figure 6B). Viability of these infected cells was about 70% at days 3 and 5 under 21% O_2_ (Figure 6A). THPM infected at an MOI of 5.0 and incubated under 21% O_2_ were completely overcome by *Mtb* multiplication by day 3 (data not shown).

Viability of PBMC-derived macrophages (infected and uninfected) in the adhered monolayer was about 97% at 3 days and 92 % at 5 days under 1% O_2_ at both MOIs. At 3 and 5 days under normoxia, about 95% of the adhered human macrophages (infected and uninfected) were viable. The total viable cell counts (by Trypan Blue dye exclusion method) of adhered hypoxic human macrophages at 3 and 5 days were about 30% of the 0-day count of 5×10^5^ macrophages per well of a 12-well plate and the respective counts for normoxic samples were about 45% of the 0-day count.

We determined the rate of *Mtb* multiplication within macrophages under 21% O_2_ and 1% O_2_. After normalization to the respective THPM cell counts, *Mtb* CFUs inside THPM under 1% O_2_ at day 5, increased to about 5-fold of 0-day values. In contrast, *Mtb* CFUs inside normoxic THPM increased to about 30-fold of 0-day values by day 5 (Figure 6C). *Mtb* CFUs in the extra-cellular medium were much lower than those inside adhered THPM monolayer (data not shown). *Mtb* replication within PBMC-derived macrophages under hypoxia was even more restricted than that inside hypoxic THPM. At day 5 under hypoxia, *Mtb* CFUs in PBMC-derived macrophages normalized to macrophage cell count was about 3-fold of 0-day values. In contrast, *Mtb* CFUs increased to about 34-fold of 0-day values at day 5 inside human PBMC-derived macrophages incubated under normoxia.

### *Mtb* inside lipid-loaded macrophages develops antibiotic resistance

If the microenvironment inside hypoxic lipid-loaded macrophages mimics what happens in the hypoxic environment of the granuloma, we might expect *Mtb* within such macrophages to develop phenotypic drug resistance which is a key indicator of dormancy [2], [4], [15]. To test for this possibility, we examined whether such phenotypic tolerance may be developed by *Mtb* within THPM and inside human PBMC-derived macrophages under 1% O_2_. At 0, 3 and 5 days after infection, *Mtb* cells inside macrophages were exposed to antibiotic for 2 additional days, under the same conditions, prior to lysis of the host cells and recovery of the bacilli. The antibiotic resistance, as a percentage of untreated control incubated for the same time-period under the same oxygen concentration, was determined by CFU determination after agar plating. As shown in Table 3, we found that phenotypic tolerance of Rif and INH of *Mtb* recovered from hypoxic THPM increased with time and reached maximal levels by 5 days under 1% O_2_ when about 8% of the total *Mtb* population was resistant to 5 µg/ml Rif and about 49% was resistant to 0.8 µg/ml INH. Further incubation (upto 16 days) in 1% O_2_ decreased the percentage of antibiotic-resistant *Mtb* (data not shown). In contrast, *Mtb* inside normoxic THPM did not develop phenotypic tolerance of the antibiotics (data not shown). *Mtb* inside human PBMC-derived macrophages incubated under hypoxia also developed phenotypic tolerance to Rif and INH, as observed in THPM (Table 3). Phenotypic resistance to Rif and INH increased to 18% and 43% respectively at day 7 inside hypoxic PBMC-derived macrophages. In contrast, *Mtb* inside normoxic human macrophages showed much lower phenotypic tolerance to Rif (4%) and negligible phenotypic tolerance (0.5%) to INH at day 7 under normoxia. Log-phase *Mtb* cultures used for infection and *Mtb* recovered from macrophages after 4 h infection and treated *in vitro* with antibiotics under normoxia for 2 days showed no resistance to Rif and INH. Thus, *Mtb* developed phenotypic drug tolerance in hypoxic THPM as well as in hypoxic human PBMC-derived macrophages.

**Table 3:**
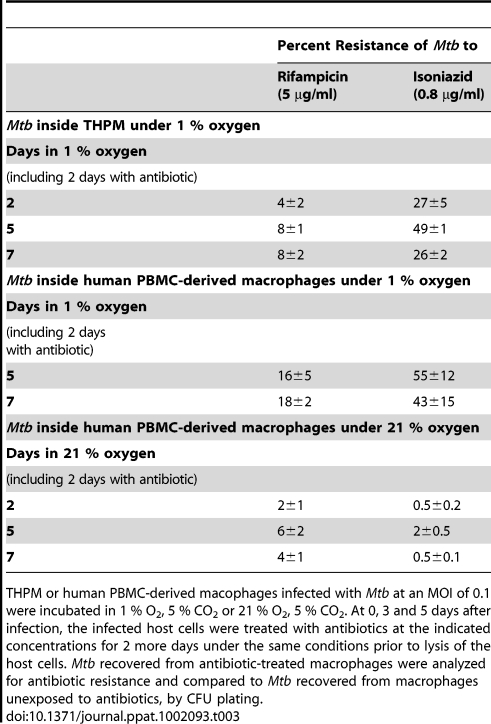
*Mtb* within hypoxic lipid-loaded macrophages develops phenotypic tolerance to antibiotics.

THPM or human PBMC-derived macophages infected with *Mtb* at an MOI of 0.1 were incubated in 1 % O_2_, 5 % CO_2_ or 21 % O_2_, 5 % CO_2_. At 0, 3 and 5 days after infection, the infected host cells were treated with antibiotics at the indicated concentrations for 2 more days under the same conditions prior to lysis of the host cells. *Mtb* recovered from antibiotic-treated macrophages were analyzed for antibiotic resistance and compared to *Mtb* recovered from macrophages unexposed to antibiotics, by CFU plating.

### *Mtb* inside hypoxic lipid-loaded macrophages accumulates neutral lipid bodies and loses acid fastness

**Figure 7:**
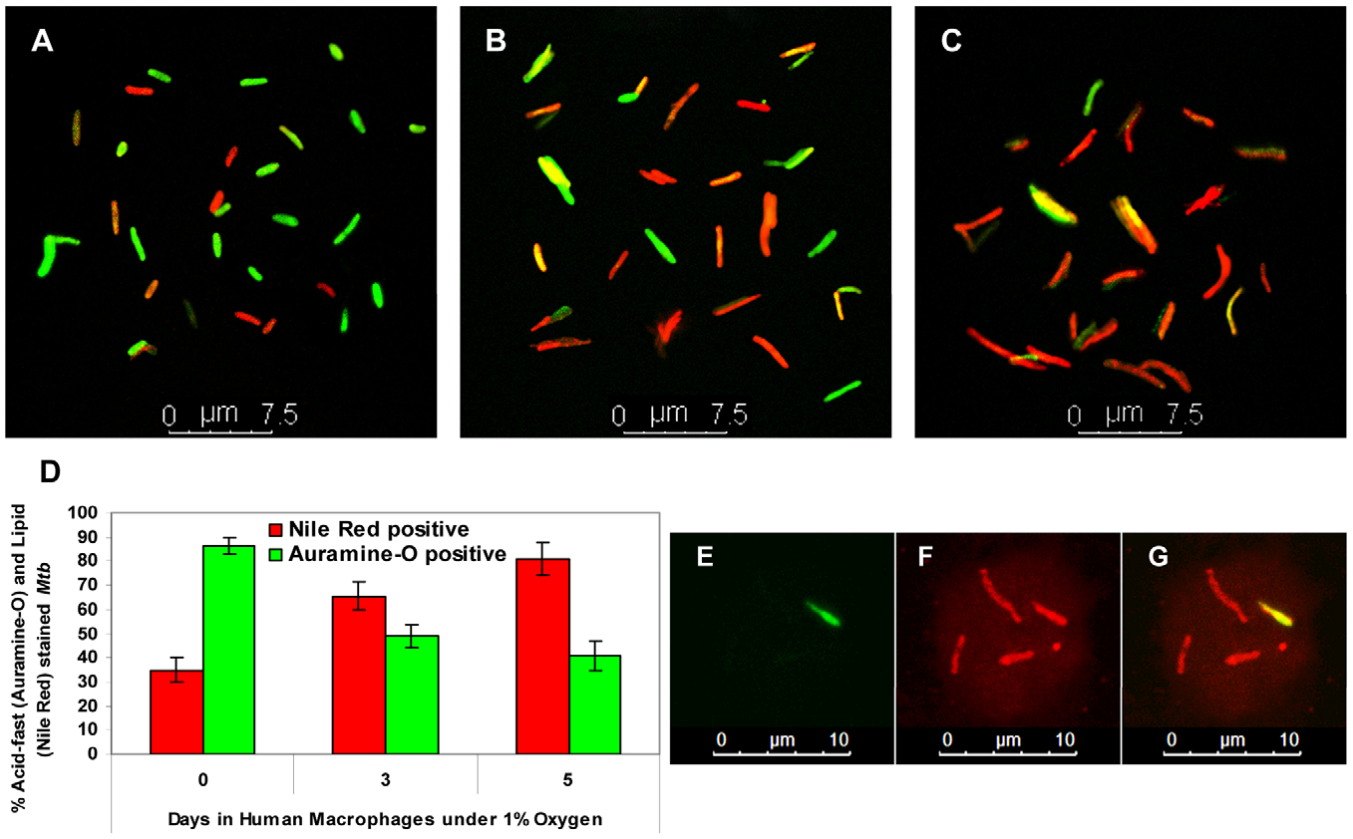
*Mtb* inside hypoxic macrophages loses acid fastness and accumulates lipid droplets. **A**–**C**, Decrease in green, Auramine-O staining, acid-fast positive *Mtb* and increase in Nile Red staining, neutral lipid-containing *Mtb* population recovered from hypoxic human macrophages with time. Human macrophages were infected at MOI 0.1 and were incubated at 1% O_2_, 5% CO_2_ at 37°C. *Mtb* cells were recovered from human macrophages after 4 h infection (**A**), at 3 days (**B**) and 5 days (**C**) and stained with Auramine-O and Nile Red; **D**, Quantitation of acid-fast and neutral lipid staining *Mtb* recovered from hypoxic human macrophages (shown in **A**–**C**) indicates a decrease in acid-fastness and increase in lipid droplet staining with time. About 250 *Mtb* cells from multiple microscopic fields were counted for enumerating green and red cells. **E**–**G,** *Mtb* within intact hypoxic THPM at day 5 showing loss of acid-fastness (green Auramine-O stain negative) and accumulation of lipid bodies (Nile Red stain positive) by confocal laser scanning microscopy. Infected THPM were subjected to 1% O_2_, 5% CO_2_ for 5 days at 37°C. Sequential laser scanning was done for Auramine-O (**E**) and for Nile Red (**F**); **G**, Merged projection of **E** and **F**.

It has been established previously that dormant *Mtb* loses acid-fast staining and accumulates Nile Red-staining lipid droplets [13], [15], [16], [25]. In order to determine whether such a phenotype is developed by *Mtb* inside hypoxic lipid-loaded macrophages, *Mtb* cells recovered from human PBMC-derived macrophages after 0, 3 and 5 days in 1% O_2_ were stained with Auramine-O and Nile Red. We observed that, in addition to the bacilli that stained with either stain, there was a subset of bacilli in the total population that retained both stains. The fraction of the *Mtb* population that stained with the green acid-fast stain (Auramine-O) decreased from about 86% at 0-day to about 40% at day 5. In contrast, *Mtb* cells that stained red with the lipid stain (Nile Red) increased with time from about 35% at 0-day to about 81% at 5-day inside hypoxic human macrophages (Figure 7A–D). Thus, by day 5 inside hypoxic macrophages, the fraction of acid-fast staining bacilli in the *Mtb* population decreased to half the level of the 0-day control, while the fraction that stained with Nile Red increased more than two-fold. Moreover, at day 5 inside hypoxic macrophages, *Mtb* cells were markedly elongated in shape when compared to the 0-day controls. In order to stain *Mtb* inside intact host cells, infected THPM after 5 days in 1% O_2_ were fixed with 4 % paraformaldehyde and stained with Auramine-O followed by Nile Red. *Mtb* cells inside such intact THPM showed loss of acid-fastness and accumulation of Nile Red staining lipid droplets similar to the *Mtb* cells that were recovered from the macrophages before staining (Figure 7E–G).

### Genes associated with dormancy and lipid metabolism are upregulated in *Mtb* within THPM

**Figure 8:**
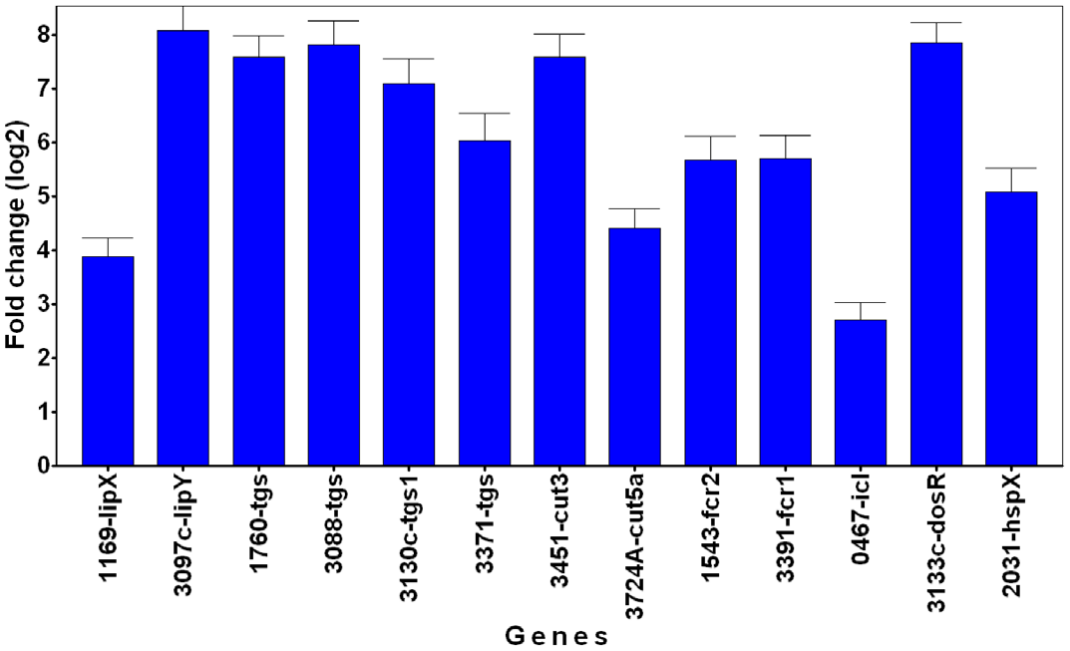
Dormancy and lipid metabolism genes are upregulated in *Mtb* recovered from lipid-loaded macrophages. TaqMan real-time PCR was used to measure the transcript levels of *Mtb* genes reported to be highly upregulated in a meta-analysis of *Mtb* microarray data from experimental models that mimicked dormancy. *Mtb* was recovered from lipid-loaded host cells at 72 h after incubation under hypoxia (1% O_2_; 5% CO_2_). Total RNA was reverse transcribed, the resulting cDNA was pre-amplified by multiplex-PCR with multiple *Mtb* gene-specific primers and the pre-amplified product was used in quantitative (q) PCR. Data was analyzed by ‘GenEx’ qPCR data analysis software (MultiD Analyses AB, Sweden) and gene transcript level was expressed as fold change in log2 scale relative to the sample from 18 h time point following normalization with 16S-rRNA as the reference gene. Average ± standard deviation from three replicates shown (n = 3); p<0.05, 18 h vs 72 h. *lip*, lipase, *tgs,* triacylglycerol synthase, *cut*, cutinase, *fcr*, fatty acyl-CoA reductase, *icl*, isocytrate lyase, *dosR*, dormancy response regulator, *hsp*, heat shock protein. The number prefixes are gene locus tag (Rv) numbers for respective *Mtb* genes.

We examined the changes in transcript levels of selected *Mtb* genes that have been shown to be upregulated in a variety of *in vitro* and *in vivo* experimental models that mimicked dormancy [27]. The gene for isocitrate lyase (*icl*) was induced (Figure 8), consistent with the idea that the pathogen in THPM utilizes fatty acids as the energy source. Induction of dormancy- and stress-responsive genes, *dos*R (*Rv3133c*) and *hspX* (*Rv2031c*), implicates the attainment of the dormant state by *Mtb* inside hypoxic, lipid-loaded THPM. In our hypoxic THPM model, *tgs1* (*Rv3130c*), *Rv3088* (*tgs4*), *Rv1760*, *Rv3371* and *Rv3087* (data not shown for this gene) were found to be highly up-regulated at 72 h after infection. It is noteworthy that *lip*Y, that was previously reported to be involved in TAG mobilization [28], was highly induced. Induction of other lipase and cutinase-like genes suggests their possible involvement in the hydrolysis of host lipids. The fatty acyl-coenzyme A reductase (*fcr*) genes *Rv3391* and *Rv1543*, that are involved in wax ester biosynthesis ([15], unpublished results) were also upregulated.

## Discussion

*Mtb* can persist for decades inside the human body in the dormant state and reactivate when the host's immune system weakens [4]. HIV infection increases the risk of reactivation leading to the deadly synergy between AIDS and TB [3], [29], [30]. Currently, there is no drug that can kill latent TB and the development of such antibiotics is critical to the cure and eradication of the disease [2], [31]. Novel drugs that target dormancy-specific metabolic pathways may enable the treatment of patients with multi- and extremely-drug resistant *Mtb* and drastically shorten the currently used, very long-term treatment period to cure TB. Understanding of dormancy-specific processes and a model system to test for inhibition of such processes are required to discover such drugs.

The pathogen is likely to go into a dormant state within macrophages that are in the hypoxic environment of the granuloma [15], [32], [33]. Such macrophages might be loaded with TAG-containing lipid bodies [6], [7], [18]. Since one of our objectives was to develop an *in vitro* model that mimics the *in vivo* situation and is suitable for high-throughput screening, we used THPM as host cells in order to avoid the well known donor-to-donor variations in primary human macrophages and the technical difficulties involved in obtaining large, homogenous populations of alveolar macrophages for experimental purposes. We validated our results obtained with hypoxic THPM by demonstrating similar observations in human macrophages which were derived from mononuclear cells isolated from the peripheral blood of healthy volunteers and subjected to hypoxia. THPM, which are capable of lipid accumulation, were reported to faithfully model the apoptotic response of human alveolar macrophages in response to *Mtb* infection [34], [35]. Furthermore, the antimycobacterial activity of INH in THPM was similar to that in human monocyte-derived macrophages [36].

The assumption that *Mtb*-infected human alveolar macrophages most likely reach a hypoxic environment within the granuloma serves as the basis for the well-studied *in vitro* hypoxic model of *Mtb* dormancy [33]. Moreover, oxygen concentrations in healthy tissue within the human body are thought to range between 5 to 71 Torr and are well below the oxygen concentration of 157 Torr in ambient room air [20], [21]. The oxygen tension in caseous granulomas of rabbits was measured to be approximately 2 Torr (∼0.3 % O_2_) [19]. Hypoxic, lipid-loaded macrophages may provide a lipid-rich sanctuary for *Mtb* during its dormancy. The killing of *Mtb* by macrophages inside the hypoxic regions of the granuloma is likely to be severely inhibited since superoxide and NO production by macrophages are greatly diminished by hypoxia [21], [37]. Furthermore, electron paramagnetic resonance-based measurements have shown that oxygen concentration in the intraphagosomal compartment was significantly lower than the extracellular environment [22]. However, macrophages infected *in vitro* with *Mtb* are currently incubated in normoxic environments where the oxygen level is far higher than that encountered by *Mtb*-infected macrophages inside the human lung granuloma. Consequently, *Mtb* inside those macrophages are not subjected to the hypoxic stress encountered inside the granuloma and do not develop phenotypic tolerance of antibiotics such as Rif and INH [36], [38] which is a key indicator of dormancy [2], [4], [15]. In order to mimic the hypoxic micro-environment within the granuloma, we infected macrophages with *Mtb* and incubated them in a 1% O_2_, 5% CO_2_ environment. Under such conditions, infected and uninfected macrophages accumulated Oil Red O-staining lipid droplets containing TAG. The replication of *Mtb* within such hypoxic lipid-loaded macrophages was greatly inhibited suggesting that a subset of the *Mtb* inside macrophages incubated under hypoxia may be entering a non-replicating state. Interestingly, hypoxia (1% O_2_) was recently shown to prolong the survival of human macrophages and the cells were reported to be adopting a glycolytic metabolism under the hypoxic conditions [39].

We postulate that host lipids may be hydrolyzed by *Mtb* lipases and the released fatty acids may be imported and re-esterified into *Mtb* TAG by the action of *Mtb tgs* gene products. The deletion of *Mtb tgs1* gene, which encodes the major TAG biosynthetic enzyme of *Mtb* [11], [14], resulted in a severe decrease of radiolabeled TAG accumulation by *Mtb* inside lipid-loaded THPM. *Mtb* inside lipid-loaded macrophages utilized host TAG that had been metabolically labeled with the fluorescent fatty acid BODIPY 558/568 C_12_ to accumulate fluorescent lipid droplets. Analysis of deconvoluted, Z-stacked fluorescence microscope images of *Mtb* recovered from fluorescent fatty acid-labeled THPM confirmed that the fluorescent lipid droplets are indeed inside the bacterial cell. Deletion of *tgs1* drastically reduced fluorescent lipid droplet accumulation and supported the finding from the radiolabeling experiments suggesting that TGS1 is a major contributor to TAG synthesis within *Mtb*. TGS1, which has very recently been shown to be associated with lipid droplets in the mycobacterial cell along with TGS2 [40], is most likely involved in *Mtb* lipid droplet synthesis. Since TAG accumulation in the *tgs1* mutant was not totally abolished, the other *Mtb tgs* gene products might also be able to contribute to TAG synthesis within *Mtb* inside the host in the absence of *tgs1*.

In order to assess whether *Mtb* inside THPM imported intact host TAG or hydrolyzed the host TAG and imported the fatty acids released, we metabolically labeled the TAG in THPM using dual-isotope labeled triolein. The glycerol backbone of the triolein was radiolabeled with ^3^H and the esterified fatty acids were labeled at the carboxyl end with ^14^C. By comparing the ^3^H:^14^C ratios of TAG isolated from *Mtb* recovered from such dual-isotope labeled THPM with that of host TAG, we were able to conclude that the main mechanism by which host lipids are used to accumulate TAG within the pathogen involves the use of fatty acids released from host TAG for resynthesis of TAG within *Mtb*. Thus, *Mtb* gene products that are involved in the import of host-derived fatty acids and synthesis of TAG within *Mtb* may play critical roles in the energy metabolism of dormant *Mtb*. We cannot, however, rule out the possibility that the import of intact TAG might also make a contribution to TAG accumulation by *Mtb* inside the host.

To determine whether host TAG-derived fatty acids were incorporated intact into TAG in *Mtb* within THPM or whether degradation of host-derived fatty acids and resynthesis of fatty acids contributed to lipid accumulation in *Mtb,* we labeled THPM TAG with [9,10-^3^H, 1-^14^C] labeled oleic acid. We observed that host TAG-derived fatty acids were being incorporated intact into *Mtb* TAG. Furthermore, if acetate derived from the catabolism of host TAG-derived fatty acids was used in the synthesis of fatty acids within *Mtb*, TAG of *Mtb* recovered from THPM should contain C_26_ fatty acid, a characteristic product of the *Mtb* fatty acid synthase [41]. The fatty acid composition of unlabeled *Mtb* TAG was identical to host TAG and C_26_ fatty acid was not detected in the TAG of *Mtb* recovered from THPM. Both the dual-isotope labeling experiments and fatty acid composition analysis of *Mtb* TAG, indicate that fatty acids released from host TAG were incorporated intact into *Mtb* TAG. The TAG levels in *Mtb* recovered from radiolabeled human macrophages were lower than that in *Mtb* recovered from THPM (Figure 2A). Possibly, inside hypoxic human macrophages, *Mtb* replication and metabolism is restricted more severely than in hypoxic THPM resulting in lower TAG synthesis by *Mtb*. This possibility correlates with our observation that shows greater antibiotic tolerance by *Mtb* in hypoxic human macrophages than in hypoxic THPM (Table 3). The quantity of TAG inside *Mtb* does not correspond directly with macrophage TAG levels probably because the quantity of TAG in the host is several orders of magnitude higher than that in the pathogen.

The accumulation of neutral lipids, loss of acid-fastness and development of phenotypic antibiotic tolerance by *Mtb* are thought to be key indicators of dormancy [2], [11], [15], [24], [25]. We observed that a subset of the *Mtb* population within hypoxic, lipid-loaded macrophages accumulated neutral lipid droplets and lost acid-fast staining indicating their dormant state. The loss of acid-fastness by a subpopulation of *Mtb* inside hypoxic macrophages supports our hypothesis that *Mtb* cells inside the lipid-loaded macrophages enter a dormant state. *Mtb* recovered from hypoxic, lipid-loaded macrophages showed phenotypic resistance to killing by Rif and INH. The natural heterogeneity of the *Mtb* population within macrophages probably prevents the entire population from displaying a uniform dormancy phenotype. This is one of the possible causes for only a subset of the *Mtb* becoming tolerant to antibiotics and accumulating storage lipids. The drastically slowed replication rate of *Mtb* inside macrophages under hypoxia probably causes the observed phenotypic antibiotic resistance. We do not have a clear understanding of the reasons for our observation of a smaller percentage of *Mtb* recovered from hypoxic macrophages showing resistance to Rif in comparison to INH. Possibly, among the non-replicating, INH-resistant *Mtb* population, a subset of the *Mtb* is metabolically inactive and thus displays Rif-resistance. The earlier findings by Peyron et al showing that only a subset (19%) of the bacilli were translocated into the lipid bodies of the foamy macrophages inside *in vitro* granulomas and exhibited intracellular lipophilic inclusions [6] could offer another reason for our observations which show a subset of *Mtb* becoming phenotypically drug tolerant.

Alternatively, TAG accumulation and phenotypic antibiotic tolerance may be independent indicators of *Mtb* dormancy. Preliminary results from our ongoing studies assessing drug tolerance of *Mtb* wild-type and *Mtb tgs1*-deletion mutant in hypoxic, lipid-loaded THPM indicate that the loss of *tgs1* causes a small, but detectable decrease in antibiotic tolerance suggesting that TAG accumulation and phenotypic drug tolerance may be independent indicators of dormancy (our unpublished observations). In our earlier findings with the *in vitro* multiple-stress model, TAG accumulation and drug tolerance appeared to be strongly correlated [15]. A major difference between the two *in vitro* dormancy models is that *Mtb* inside the lipid-loaded macrophages is exposed to a readily available supply of host TAG in the lipid bodies whereas in the multiple stress model *Mtb* was cultured in a nutritionally limited environment (10% Dubos medium). It appears that the two indicators of dormancy, TAG accumulation and drug tolerance, show strong correlation only when *Mtb* experiences nutritionally limiting conditions. We found that at day 5 after infection, the antibiotic resistance of *Mtb* recovered from hypoxic lipid-loaded macrophages reached maximal levels. In normoxic macrophages, *Mtb* did not develop drug tolerance to the high levels seen in hypoxic, lipid-loaded macrophages (Table 3) probably due to the high rate of multiplication (Figure 6C). This finding is consistent with earlier reports that showed a lack of antibiotic resistance in *Mtb* inside normoxic macrophages [36], [38]. Thus, unlike in the conventional macrophages incubated under normoxia (our results and [36], [38]), in the hypoxic, lipid-loaded macrophages, *Mtb* displays intracellular TAG accumulation, phenotypic drug tolerance and loss of acid-fastness - the three key indicators of dormancy. A recent report showed that human macrophages infected with *Mycobacterium leprae* secreted TLR2 and TLR6 and caused uninfected macrophages to become lipid-loaded in addition to the infected macrophages [42]. It would be interesting to examine in future studies whether such paracrine signaling mechanisms play similar roles in hypoxic macrophages.

We examined the transcript levels of selected *Mtb* genes involved in lipid metabolism and known to be up-regulated in a meta-analysis of *Mtb* microarray data from *in vitro* and *in vivo* experimental models that mimicked dormancy [27]. Interestingly, the *tgs*, *lip*, *cut* and *fcr* genes that received high up-regulation scores in the meta-analysis [27], were also found to be significantly induced in our hypoxic lipid-loaded THPM model. Several *tgs* genes, including *tgs1*, were induced indicating their possible involvement in the storage of fatty acids derived from host lipids as TAG within the pathogen, consistent with our hypothesis. The other *tgs* genes induced in this system might be responsible for the finding that TAG accumulation was not totally abolished in the *tgs1* mutant. We reported previously that the *Mtb* lipase (LIPY), which belongs to the hormone-sensitive lipase family, was capable of releasing fatty acids from TAG stored within the pathogen for utilization during starvation [28]. LIPY has subsequently been shown by others to be localized on the mycobacterial cell wall and plays a major role in the hydrolysis of TAG within *Mtb* [43], [44]. The upregulation of the *lipY* gene in *Mtb* recovered from THPM is consistent with the observation that the TAG that accumulates in the pathogen is generated within *Mtb* from fatty acids released from host TAG and suggests its possible involvement in releasing fatty acids from host TAG. However, further studies are needed to directly prove this potential role of LIPY. The induction of *Rv1543*/ *fcr*2 and *Rv3391*/ *fcr*1, that we have identified as the two fatty acyl-coenzyme-A reductase genes involved in wax ester synthesis in *Mtb* (unpublished results), is consistent with the wax ester synthesis observed in *Mtb* recovered from lipid-loaded THPM. We have previously reported that the transcripts of these two *fcr* genes were upregulated in *Mtb* subjected *in vitro* to multiple stress that caused accumulation of wax esters [15]. The induction of *icl,* which is critical for the utilization of fatty acids by the pathogen inside the host cell, supports our hypothesis that *Mtb* inside lipid-loaded macrophages utilizes host TAG-derived fatty acids as the main energy source during dormancy. This is the first report on gene expression changes in *Mtb* within hypoxic lipid-loaded macrophages. A previous transcriptome analysis of *Mtb* inside normoxic macrophages provided information on the gene transcription in *Mtb* at very early stages of infection and did not address the changes that occur during latency in the hypoxic lipid-loaded macrophages found in granuloma [45]. In the normoxic macrophage model only two *tgs* genes (*Rv3087* and *Rv3088*) were reported to be up-regulated by approximately 4 to 5 fold at 24 h of infection compared to the *in vitro* grown *Mtb* cells [45]. It is likely that the gene expression changes we report are more relevant to those experienced by the pathogen inside the hypoxic environments of the human granuloma.

The role of foamy macrophages as a nutrient-rich reservoir for *Mtb* in the TB granuloma was proposed in a report by Peyron et al who showed that *Mtb* induced the formation of foamy (lipid-loaded) macrophages in the *in vitro* granuloma model developed by the same authors earlier [6], [46]. Furthermore, the authors demonstrated that oxygenated mycolic acids play a central role in the maturation of macrophages into lipid-loaded macrophages and *Mtb* cells within the foamy macrophages were shown to persist in a dormant non-replicative state [6]. Our results, which demonstrate that a subset of the *Mtb* population inside hypoxic human lipid-loaded macrophages displays phenotypic antibiotic tolerance, correlate well with these earlier findings on *Mtb* dorfmancy inside the foamy macrophages of *in vitro* granulomas by Peyron et al. However, in contrast to the above *in vitro* granuloma model, we observed that, under hypoxia, macrophage lipid bodies containing TAG were formed in the absence of *Mtb* infection suggesting that oxygenated mycolic acids probably do not play a major role in lipid body formation in host cells under hypoxia (Figure 1D, E). Since TAG levels in hypoxic macrophages infected with *M. smegmatis* were slightly lower than *Mtb*-infected macrophages, the presence of oxygenated mycolic acids appears to mildly stimulate TAG formation in host lipid bodies under hypoxia. But, for reasons unclear to us, we could not observe significant differences in macrophage TAG accumulation between uninfected, *Mtb*-infected and *M. smegmatis*-infected cells in our normoxic samples (Figure 1D,E).

Our findings here are in agreement with the earlier report by Bostrom et al, which served as the conceptual basis for our hypoxic human macrophage model, showing lipid droplet accumulation in uninfected human macrophages under hypoxia [18]. Interestingly, Peyron et al observed that a subset of the bacilli inside foamy macrophages were translocated into the host lipid bodies and exhibited electron-translucent intracellular lipophilic inclusions at day 11 post-infection. *Mtb* cells which come into such direct contact with host lipid bodies most likely import fatty acids derived from host TAG, which is the major constituent of the lipid bodies [47], and sequester a portion of the fatty acids in *Mtb* TAG, as our results show. Inside hypoxic lipid-loaded macrophages, host TAG-derived fatty acids are also used by the *Mtb* cells for the synthesis of polar lipids and wax esters as seen in our results (Figure 4). The free fatty acids we detected inside *Mtb* isolated from lipid-loaded macrophages (Figure 4) likely provide metabolic energy to *Mtb* since it has been well established that the pathogen isolated from the host prefers fatty acids as an energy source [8], which is also suggested by the upregulation of the isocitrate lyase gene of *Mtb* observed by us (Figure 8). The host TAG-derived fatty acids appear to be utilized immediately by *Mtb* in polar lipid and wax ester biosynthesis apart from *Mtb* TAG synthesis (Figure 4). However, we postulate that the TAG that is synthesized within *Mtb* from host fatty acids is probably not for the purpose of immediate utilization but stored as an energy source for utilization during dormancy and subsequent reactivation of *Mtb*. Further experimentation is needed to prove this postulate.

Further studies are also needed to identify *Mtb* gene products that function in the import of fatty acids released from host TAG. Such gene products may prove to be attractive targets for novel drugs against the dormant pathogen. Our novel model of *Mtb* dormancy can be used to better understand the metabolic pathways critical for the pathogen as it enters the dormant state and can be adapted for high-throughput screening to discover drug candidates that can kill dormant *Mtb* and thus help in the cure and eradication of tuberculosis.

## Materials and Methods

### Ethics statement

Human blood was collected at a blood donation center of the Florida Blood Centers from healthy volunteers as per written informed consent. Florida Blood Centers operate under license from the Food and Drug Administration of the US Department of Health and Human Services. Therefore, the use of blood from this source is exempt from our institutional review board.

### Cell culture and *Mtb* infection

The buffy coat provided by the Florida Blood Centers after separation of other blood components was used for isolation of peripheral blood mononuclear cells (PBMCs) by density gradient centrifugation on Ficoll-Paque PLUS (GE Healthcare, Piscataway, NJ), following previously described procedures [48]. PBMCs were resuspended in RPMI-1640 and allowed to adhere onto plastic petri dishes or multi-well plates and non-adherent cells were removed by gentle washes with phosphate-buffered saline (PBS) after 2 h. Adherent PBMCs were then allowed to differentiate to macrophages over a period of 7 days under 21% O_2_, 5% CO_2_ atmosphere in RPMI-1640 containing 10% (v/v) human serum AB (Lonza Walkersville Inc., Walkersville, MD) in the presence of 10 ng/ml granulocyte-macrophage colony stimulating factor (GM-CSF) (Sigma, St. Louis, MO), as described by others [48], [49]. PBMCs differentiated under such conditions were reported to display an alveolar macrophage-like phenotype [49]. THP-1 cells were cultured in RPMI 1640 (ATCC, Manassas, VA) supplemented with 10% fetal calf serum in a 5% CO_2_ atmosphere at 37°C and differentiated into THPM by stimulation with 100 nM phorbol 12-myristate 13-acetate for 3 days [36]. Human macrophages and THPM were counted, after trypsinization, at the specific time-points.

*Mtb* H37Rv and *Mycobacterium smegmatis* were grown in Middlebrook 7H9 medium (supplemented with 10% OADC, 0.2% glycerol and 0.05% Tween 80) to an OD_600_ of 0.7, sonicated and used to infect the macrophages obtained above for 4 h at 37°C under 21% O_2_, 5% CO_2_ atmosphere in RPMI-1640 containing 10% serum. The multiplicity of infection (MOI) used was either 0.1 or 5 bacilli per macrophage. Extracellular *Mtb* bacilli were removed by washing the infected cells thrice with PBS after which the macrophages were incubated in RPMI-1640 containing 10% serum at 37°C under hypoxia (1% O_2_, 5% CO_2_) in a Hera Cell 150 CO_2_ incubator with O_2_ control (Thermo Fisher Scientific, Waltham, MA).

### Radioisotope labeling of macrophages

Human macrophages were metabolically labeled with [1–^14^C]oleic acid (60 mCi/mmol; 8–10 µCi/ 4×10^6^ macrophages) and THPM were metabolically labeled with [9,10–^3^H]oleic acid (60 Ci/mmol; 8–10 µCi/ 7×10^6^ THPM) or [1–^14^C]oleic acid (60 mCi/mmol; 8–10 µCi/ 7×10^6^ THPM) under 1% O_2_ for 24 h. THPM were also metabolically labeled using double isotope labeled triolein [glycerol-1,2,3-^3^H (60 Ci/mmol; 20 µCi/ 7×10^6^ THPM), carboxyl-1-^14^C (55 mCi/mmol; 40 µCi/ 7×10^6^ THPM)] or double isotope labeled oleic acid [9,10–^3^H (60 Ci/mmol; 8–10 µCi/ 7×10^6^ THPM), 1–^14^C (60 mCi/mmol; 8–10 µCi/ 7×10^6^ THPM)]. Radiolabeled chemicals were obtained from American Radiolabeled Chemicals, Inc. (St. Louis, MO).

### Lipid analysis

The analysis of total lipid accumulation in the host cells was performed with 1.8×10^7^ THP-1 cells seeded per 150 mm plate and differentiated to THPM as described above, for each data point collected. THPM were infected with *Mtb* at an MOI of 0.1 and extracellular *Mtb* bacilli were removed with PBS washes. Infected THPM and uninfected controls were incubated under hypoxia or normoxia for the indicated time-periods. For experiments with radiolabeled lipids, 7×10^6^ THP-1 were seeded per 100 mm plate and differentiated into THPM for every data point collected. Alternatively, human PBMCs were differentiated into about 4×10^6^ macrophages per 100 mm plate after 7 days for every data point collected. Following radio-labeling as described above, host cells were washed with PBS to remove unincorporated radiolabels before infection with *Mtb* at an MOI of 5.0 and incubated in 1% O_2_. After incubation in the indicated oxygen concentration, extracellular medium was removed and the adhered macrophages were lysed in water containing Triton X-100 (0.05%, v/v), sonicated and the lysate was centrifuged at 3500 x g. The *Mtb* cells (3500 x g pellet) were washed thrice with 0.05% Triton X-100 in water and each 3500 x g pellet was treated with 10,000 U of TAG lipase from *Candida rugosa* (Sigma, St. Louis, MO) for 4 h at 37°C to remove background TAG adhering to their outer surface before lipid extraction with chloroform: methanol (2∶1, v/v). Macrophage lipids were isolated from the 3500 x g supernatant of host cell lysate by chloroform extraction following acidification. Quantitation of TAG band intensity in unlabeled total lipid extracts was done by densitometric analysis of the TAG band using an AlphaImager gel documentation system (AlphaInnotech, San Leandro, CA) after dichromate/sulfuric acid charring of the TLC plate. Dual isotope-labeled TAG was purified from the respective total lipid extracts by silica thin-layer chromatography (TLC) in hexane : diethyl ether : formic acid (40∶10∶1, by volume) as the solvent system, using authentic triolein (Sigma, St. Louis, MO) as the external reference standard. Ratios of ^3^H and ^14^C radioactivities in TAG were determined from the disintegrations per minute (dpm) calculated after liquid scintillation counting in the appropriate energy windows using a Tri-Carb 2900 liquid scintillation analyzer (Perkin-Elmer, Waltham, MA).

### Fatty acid composition analysis

After infection with *Mtb* at an MOI of 0.1, THPM were incubated under 1% O_2_ for 7 days. *Mtb* cells were isolated from THPM and treated to remove contaminating host TAG as described above. TAG from *Mtb* isolated from THPM was purified by preparative TLC. Methyl esters of fatty acids (FAMEs) were prepared from THPM and *Mtb* TAG and analyzed using a CP-TAP CB capillary column attached to a CP-3900 gas chromatograph (Varian, Inc., Palo Alto, CA) under a temperature control program. FAMEs prepared from TAG of *Mtb*-infected macrophages labeled with [^14^C]oleate and TAG from *Mtb* recovered from such macrophages were analyzed by AgNO_3_-TLC (silica gel with 10% AgNO_3_, Analtech, Newark, DE) in hexane : diethyl ether : acetic acid, 47∶2∶1, v/v/v (developed twice) as the solvent system The FAMEs from THPM and *Mtb* TAG were also analyzed by reversed-phase TLC (HPTLC RPS Uniplate, Analtech, Newark, DE) in acetonitrile: methanol: acetic acid: water, 30∶70∶5∶1, v/v/v as the solvent system.

### Fluorescent fatty acid labeling

THPM were metabolically labeled for 24 hours under 1% O_2_, 5% CO_2_ at 37°C with 5 µg/ml of the fluorescent fatty acid BODIPY 558/568 C_12_ (Invitrogen/Molecular Probes, Carlsbad, CA). The THPM were washed with PBS to remove unincorporated fluorescent fatty acid and infected with *Mtb* (wild type or Δ*tgs1* [*Rv3130c*] mutant) at an MOI of 0.1 or 0.25. After 4 h infection, the extracellular *Mtb* bacilli were removed by washing with PBS and the infected THPM were incubated under 1% O_2_, 5% CO_2_ at 37°C. After different periods of incubation up to 7 days, THPM were either collected intact by trypsinization from culture plates or lysed with Triton X-100 (0.05%, v/v in water), probe-sonicated and *Mtb* from THPM were recovered by centrifugation at 3500 x g. Intact THPM cells were centrifuged at 300 x g, resuspended in PBS and fixed with formaldehyde. *Mtb* cells were resuspended in PBS containing 0.05% Triton X-100, sonicated and fixed with formaldehyde (4%, v/v). THPM or *Mtb* cells were allowed to adhere to poly-L-lysine coated cover slips and mounted in Slow Fade (Invitrogen/Molecular Probes, Carlsbad, CA).

### Microscopy

*Mtb* cells, recovered from PBMC-derived macrophages (infected at MOI 0.1) at 3 and 5 days under hypoxia, were concentrated by centrifugation and stained with Auramine-O (TB Fluorescent Stain Kit M, Becton Dickinson, Sparks, MD) and with Nile Red (Invitrogen/Molecular Probes, Carlsbad, CA) following a previously published protocol [13] and examined by confocal laser scanning microscopy (Leica TCS SP5; Leica Microsystems, Mannheim, Germany) with Z-stacking. Scanned samples were analyzed by LAS AF software (Leica) for image projection. Intact THPM infected with *Mtb* at an MOI of 0.1 and incubated 5 days under hypoxia were fixed with 4% paraformaldehyde, stained and imaged similarly.

Microscopy for the Oil Red-O staining experiments and BODIPY-labeling experiments were performed with a Nikon TE2000 microscope (Nikon Corp., Tokyo, Japan) equipped with a Nikon 1.4 NA Plan Apo VC 100X oil-immersion objective. Images were acquired using a CoolSnap HQ^2^ camera (Photometrics, Tucson, AZ) or a Nikon Digital Sight DS Ri1 Camera. “NIS Elements” software (Nikon) was used for acquisition, measurements and deconvolution. At different periods of incubation under 1% O_2_ or 20% O_2_, intact THPM were collected from culture plates by trypsinization, fixed with paraformaldehyde, stained with Oil Red-O (0.21% w/v in 60% isopropanol) and imaged in bright field. For each field of *Mtb* cells labeled with BODIPY 558/568 C_12_, the fluorescence image using Texas Red filter set (Chroma, Rockingham, VT) and differential interference contrast (DIC) image were captured. To calculate the fluorescence intensity of single cells, the maximum pixel values of the background of the image was subtracted from the measured pixel value of each BODIPY 558/568 C_12_-containing cell. For quantitative comparison, the fluorescence of a few hundred individual cells was measured. All fluorescence images used for quantitative comparison were taken the same day at the same exposure. For intact THPM, images were taken using the Texas Red filter set and the DAPI filter set (Chroma, Rockingham, VT). When needed, image slices for deconvolution were taken at 0.2 µm.

### THPM cell counts, *Mtb* CFU and phenotypic antibiotic resistance determinations

For determining *Mtb* CFUs in THPM, 1.2×10^6^ THP-1 cells were differentiated into THPM in each well of 6-well plates and infected with *Mtb* at an MOI of 0.1 or 5.0. Uninfected and infected cells were then incubated in either 1% O_2_ or 21% O_2_. At the indicated time-points, floating THPM cells were collected by centrifugation of the medium in each well at 300 x g. *Mtb* in extracellular medium was collected by centrifugation of the 300 x g supernatant at 3500 x g. Adhered THPM were trypsinized and collected by centrifugation at 300 x g. A similar protocol was followed for the PBMC-derived human macrophages. Cell counts were determined using a hemocytometer. Cell viability was determined by trypan blue dye exclusion method. The *Mtb* CFUs in the extra-cellular medium, floating and adhered THPM populations were determined by resuspending the pellets from above in distilled water containing 0.05% Triton X-100, by vigourous vortexing and sonication in a water-bath to lyse host cells and disperse bacterial clumps, and plating serial dilutions on Middlebrook 7H10 plates followed by incubation for 28 days at 37°C.

For phenotypic antibiotic resistance determinations, macrophages were infected with *Mtb* at an MOI of 0.1. After incubation in 1% O_2_, 5% CO_2_ or 21% O_2_, 5% CO_2_ for 0, 3 or 5 days, Rif (5 µg/ml) or INH (0.8 µg/ml) was added to the infected macrofphages which were then incubated for an additional 2 days under the same conditions. Extracellular medium and floating host cells were removed and adhered macrophages were lysed in distilled water containing 0.05% Triton X-100. The *Mtb* in the lysates were analyzed for antibiotic resistance by plating on Middlebrook 7H10 agar plates without antibiotic and CFUs were determined after 28 days at 37°C. For zero-day time point, *Mtb* were recovered from host cells after 4 h infection and then treated with antibiotics in Middlebrook 7H9 medium for 2 days under normoxic conditions. Log-phase *Mtb* cultures used for infection were treated with antibiotics in Middlebrook 7H9 medium for 2 days under normoxic conditions.

### Gene expression analysis of intracellular *Mtb* - infection and RNA isolation

THPM were infected with *Mtb* at an MOI of 0.1 and incubated under hypoxia as described above. At each time point *Mtb* infected THPM were lysed in Trizol reagent (Invitrogen/ Life Technologies, Carlsbad, CA) containing 20 µg/ml linear polyacrylamide (Ambion, Austin, TX), the lysate was homogenized at high speed with 10 mm homogenizer (Omni International, Kennesaw, GA) for 5 min and centrifuged at 3500 x g to pellet *Mtb* cells. The pellet was resuspended in Trizol reagent containing 20 µg/ml linear polyacrylamide (Ambion), the suspension was placed in 2 ml tubes containing 0.5 ml of 0.1 mm Zirconia/silicon beads (Lysing matrix B, MP Biomedicals, Solon, OH) and *Mtb* cells were disrupted four times for 40 sec each at speed 6 (Fast-Prep instrument, MP Biomedicals, Solon, OH) with cooling on ice for 1 min after each cycle of burst. Further down-stream processing, RNA isolation and first strand cDNA synthesis were performed as described previously [15].

#### Multiplex Pre-amplification PCR and TaqMan Real-Time PCR

To evaluate gene expression changes of the pathogen within the THPM under hypoxia a modified pre-amplification method was followed [50]. The first strand cDNA synthesized using random hexamer primers were used for multiplex-PCR (prior to real-time PCR amplification) with selected multiple *Mtb* genes. The multiplex PCR primers were designed by Visual OMP software version 7.2 (DNA software, Inc., Ann Arbor, MI). ‘Thermo-BLAST’ module (version 1.2.22.0) of Visual OMP was used to determine the specificity of primer hybridization against the entire *Mtb* genome sequence under the same PCR reaction condition for all the targets. Each multiplex PCR primer pair was verified for specificity and efficiency in single-plex PCR reactions with genomic DNA and cDNA as templates. Advantage2 polymerase PCR reagent (Clontech, Mountain View, CA) was used for multiplex pre-amplification PCR and the PCR reaction mix contained (50 µl reaction volume) 5 µl of 10X reaction buffer, 1 µl of 10 mM dNTPs, 5 pM final concentration of primer pair mix for all the respective number of target genes (in general the aliquot for multiple-primer mix is one tenth of the number of targets), 1 µl Advantage2 DNA polymerase, 4 to 9 µl aliquot of cDNA (volume of cDNA depended on the initial amount of RNA taken into the reverse transcriptase reaction) and the final volume was made up to 50 µl with H_2_O. PCR amplification was carried out with the following cycling parameters: 95°C for 1 min followed by 15 to 20 repeats of PCR cycle of 95°C for 30 sec, 60°C for 25 sec and 68° for 1 min. This pre-amplification product was used in TaqMan real-time PCR to measure the CT (cycle threshold) values for each target gene. Nested TaqMan primer pair and probes were designed on the multiplex-PCR product sequence for each target gene with Primer Express software (Applied Biosystems / Life Technologies, Carlsbad, CA). Each TaqMan real-time PCR primer pair was checked for amplifying the unique and the right sized product using the melt-curve analysis with 7900 HT real-time PCR system and SDS2.3 software (Applied Biosystems, Life Technologies, Carlsbad, CA). The relative transcript levels for each target gene was measured by TaqMan real-time PCR with 7900 HT real-time system (Applied Biosystems, Foster City, CA). The raw CT values were exported into excel spreadsheet and analyzed by GenEx software (MultiD AB, Sweden) to determine the relative expression of each gene. 16S rRNA gene was used as the reference gene to normalize the CT values of the target genes and 18 h time point sample was used as the calibrator.

### Accession numbers

*tgs1/Rv3130c*, P0A650; *tgs2/Rv3734c*, P67210; *lipY/Rv3097c*, P77909; *Rv3391/fcr1*, O50417; *Rv1543/fcr2*, P66779; *Rv3087*, O53304; *tgs4/Rv3088*, P67208; *lipX/Rv1169c*, Q79FR5; *Rv1760*, O06795; *Rv3371*, O50400; *cut3/Rv3451*, P0A536; *cut5A/Rv3724A*, Q79FA5; *icl1/Rv0467*, P0A5H3; *dosR*/*Rv3133c*, P95193; *hspX/*Rv2031c, P0A5B7
